# Structural and Dynamic Analysis of Sulphur Dioxide Adsorption in a Series of Zirconium‐Based Metal–Organic Frameworks

**DOI:** 10.1002/anie.202207259

**Published:** 2022-07-25

**Authors:** Jiangnan Li, Gemma L. Smith, Yinlin Chen, Yujie Ma, Meredydd Kippax‐Jones, Mengtian Fan, Wanpeng Lu, Mark D. Frogley, Gianfelice Cinque, Sarah J. Day, Stephen P. Thompson, Yongqiang Cheng, Luke L. Daemen, Anibal J. Ramirez‐Cuesta, Martin Schröder, Sihai Yang

**Affiliations:** ^1^ Department of Chemistry University of Manchester Manchester M13 9PL UK; ^2^ Diamond of Light Source Harwell Science Campus Oxfordshire OX11 0DE UK; ^3^ Department of Engineering Sciences University of Oxford Oxford OX1 3PJ UK; ^4^ Neutron Scattering Division Oak Ridge National Laboratory Oak Ridge TN 37831 USA

**Keywords:** Capture, Conversion, Crystallography, Metal–Organic Frameworks, Sulfur Dioxide

## Abstract

We report reversible high capacity adsorption of SO_2_ in robust Zr‐based metal–organic framework (MOF) materials. Zr‐bptc (H_4_bptc=biphenyl‐3,3′,5,5′‐tetracarboxylic acid) shows a high SO_2_ uptake of 6.2 mmol g^−1^ at 0.1 bar and 298 K, reflecting excellent capture capability and removal of SO_2_ at low concentration (2500 ppm). Dynamic breakthrough experiments confirm that the introduction of amine, atomically‐dispersed Cu^II^ or heteroatomic sulphur sites into the pores enhance the capture of SO_2_ at low concentrations. The captured SO_2_ can be converted quantitatively to a pharmaceutical intermediate, aryl N‐aminosulfonamide, thus converting waste to chemical values. In situ X‐ray diffraction, infrared micro‐spectroscopy and inelastic neutron scattering enable the visualisation of the binding domains of adsorbed SO_2_ molecules and host–guest binding dynamics in these materials at the atomic level. Refinement of the pore environment plays a critical role in designing efficient sorbent materials.

## Graphical abstract

A series of Zr‐based metal–organic framework materials have been investigated for reversible SO_2_ uptake. The incorporation of atomically‐dispersed Cu^II^, amine or heteroatomic sulphur sites enhances the uptake of SO_2_ at low concentrations. This work confirms that control of pore environments is an important approach for optimising the adsorption of SO_2_ at low concentrations.

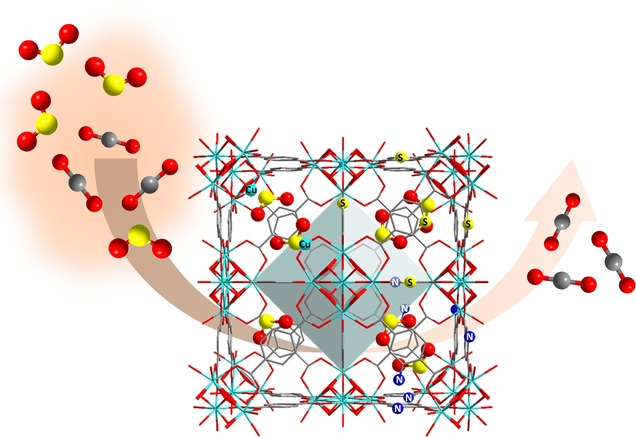

## Introduction

Sulphur dioxide (SO_2_) is an important air pollutant as well as a key chemical feedstock for the synthesis of sulfuric acid and various fine chemicals.[^1^, ^2^, ^3^, ^4^, ^5^] State‐of‐the‐art flue‐gas desulphurisation (FGD) technology uses limestone slurry to capture SO_2_ effectively, but this is an irreversible process that generates a tremendous amounts of solid waste.[^6^, ^7^] Recovery of SO_2_ from exhaust gases via reversible adsorptive techniques can promote the development of “waste‐to‐chemical” technologies, but it relies on the development of efficient sorbent materials that not only show high and reversible adsorption of SO_2_, but also are highly robust so that regeneration of the sorbent can be achieved for use over many cycles.

Metal–organic framework (MOF) materials have been studied widely for gas adsorption and separation owing to their high surface area and tuneable pore environment.[^8^, ^9^] The study of MOF materials as SO_2_ reservoirs has seen significant interest recently,[^10^, ^11^, ^12^] but only a limited number of MOFs show reversible SO_2_ uptake and structural stability upon desorption: for example, Mg‐MOF‐74 (8.6 mmol g^−1^),^[13]^ EDTA‐MOF‐808 (9.8 mmol g^−1^),^[14]^ [Ni(bdc)(ted)_0.5_] (10.0 mmol g^−1^),^[15]^ [Zn_2_(L)_2_(bipy)] (10.9 mmol g^−1^),^[16]^ SIFSIX‐1‐Cu (11.0 mmol g^−1^),^[17]^ ECUT‐111 (11.6 mmol g^−1^),^[18]^ DMOF (13.1 mmol g^−1^),^[19]^ MFM‐300(Sc)@EtOH (13.2 mmol g^−1^),^[20]^ MOF‐808 (15.3 mmol g^−1^),^[14]^ MFM‐170 (17.5 mmol g^−1^)^[21]^ and MIL‐101(Cr)‐4F(1 %) (18.4 mmol g^−1^)^[22]^ all at 298 K and 1 bar of SO_2_. MOFs constructed from {Zr_6_} clusters are renowned for their high stability.[^23^, ^24^, ^25^] However, their performance in adsorption of SO_2_ has been poorly explored and, to date, only few Zr‐MOFs have shown reversible SO_2_ adsorption at 298 K and 1 bar, including MFM‐601 (12.3 mmol g^−1^)^[26]^ and NU‐1000 (10.9 mmol g^−1^).^[27]^

Herein, we report a systematic structural and dynamic analysis of adsorption of SO_2_ in seven robust Zr‐MOFs: UiO‐66, UiO‐66‐NH_2_, UiO‐66‐Cu^II^, Zr‐DMTDC (H_2_DMTDC=3,4‐dimethylthieno[2,3‐b]thiophene‐2,5‐dicarboxylic acid), Zr‐bptc, MFM‐133 and MFM‐422. Compared with UiO‐66, the introduction of amine groups (UiO‐66‐NH_2_), thienothiophene groups (Zr‐DMTDC) or atomically‐dispersed Cu^II^ sites (UiO‐66‐Cu^II^) afford 76 %, 47 % and 43 % enhancement of SO_2_ uptake at 0.1 bar and 298 K, respectively. Zr‐bptc exhibits an exceptional SO_2_ uptake of 6.2 mmol g^−1^ at 0.1 bar and 298 K and dynamic breakthrough confirms the highly selective capture of SO_2_ from a mixture of SO_2_/CO_2_ (2500 ppm SO_2_, 15 % CO_2_ diluted in He). In addition, the captured SO_2_ in Zr‐bptc can be converted to aryl N‐amino sulphonamide, an important compound in medicinal chemistry, thus fulfilling the “waste‐to‐chemicals” target. MFM‐422 shows a high Brunauer–Emmett–Teller (BET) surface area of 3296 cm^2^ g^−1^ and an exceptional and reversible uptake of SO_2_ of 31.3 mmol g^−1^ at 1 bar and 273 K. These materials show high stability with full retention of structure and uptake capacities over multiple cycles of adsorption‐desorption of dry SO_2_. The adsorption domains and binding dynamics of SO_2_ in these MOFs have been studied by in situ synchrotron X‐ray powder diffraction (SXPD), inelastic neutron scattering (INS), and synchrotron infrared micro‐spectroscopy (microFTIR) to provide key insights into the structures and dynamics of high adsorption of SO_2_ in these systems.

## Results and Discussion

**Figure 1:**
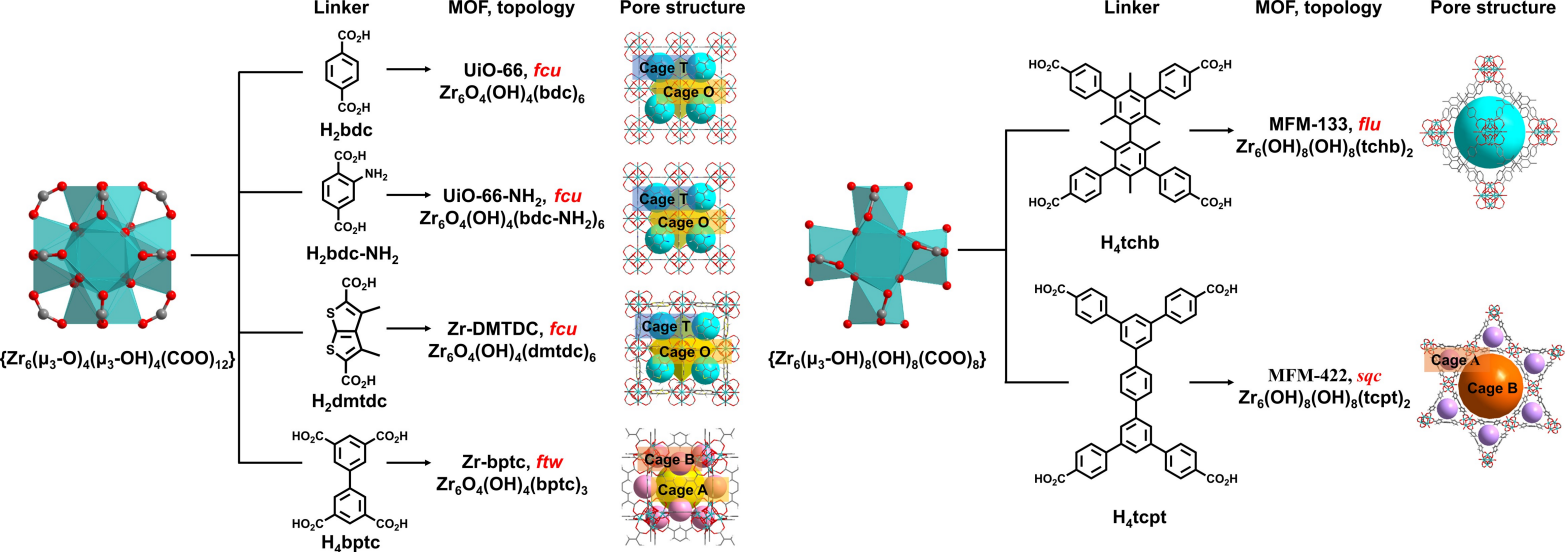
Views of {Zr_6_}‐clusters, linkers and structures of the Zr‐based MOFs used in this study (Zr, aqua; C, grey; O, red; H, white; S, yellow).

UiO‐66,^[28]^ UiO‐66‐NH_2_,^[29]^ UiO‐66‐Cu^II[30]^ and Zr‐DMTDC^[31]^ are iso‐structural and constructed from 12‐connected {Zr_6_(μ_3_‐O)_4_(μ_3_‐OH)_4_(OOCR)_12_} clusters bridged by dicarboxylates to give cubic structures of ***fcu*** topology (Figure 1). These structures consist of two types of cages with an octahedral cage (Cage O, diameter of 9–12 Å) connecting to eight tetrahedral cages (Cage T, diameter of 7.3 Å) via triangular faces (Figure 1). The pores of UiO‐66‐NH_2_ and Zr‐DMTDC are decorated with free −NH_2_ and −S− sites, respectively, affording additional binding sites for guest molecules. In UiO‐66‐Cu^II^, defect sites with free −OH/−OH_2_ sites in the pore are decorated with open Cu^II^ sites. Desolvated UiO‐66, UiO‐66‐NH_2_, UiO‐66‐Cu^II^ and Zr‐DMTDC show BET surface areas of 1221, 1037, 1068 and 1345 m^2^ g^−1^, respectively.

Zr‐bptc is built from 12‐connected {Zr_6_(μ_3_‐O)_4_(μ_3_‐OH)_4_(OOCR)_12_} clusters and tetracarboxylate ligands in an open framework of ***ftw*** topology.^[32]^ Desolvated Zr‐bptc consists of cubic cages (cage A) of diameter 12 Å fused to tetrahedral cages (cage B) of 4.5 Å diameter (Figure 1) with a BET surface area of 960 m^2^ g^−1^. MFM‐133^[33]^ is constructed from 8‐connected {Zr_6_(OH)_8_(OH)_8_(OOCR)_8_} clusters and thcb^4−^ ligands (H_4_thcb=3,3′,5,5′‐tetrakis(4‐carboxyphenyl)‐2,2′,4,4′,6,6′‐hexamethyl‐1,1′‐biphenyl) to form a ***flu*** topology. MFM‐133 shows an axially elongated octahedral cage (10.4×10.4×25.9 Å) and a BET surface area of 2156 m^2^ g^−1^ (Figure 1). A new MOF, MFM‐422, is constructed by linking 8‐connected {Zr_6_(OH)_8_(OH)_8_(OOCR)_8_} clusters with the tetratopic ligand 3,3′′,5,5′′‐tetrakis(4‐carboxyphenyl)‐*p*‐terphenyl (H_4_tcpt) to give a neutral framework of ***sqc*** topology. MFM‐422 is comprised of a trigonal cage (cage B, diameter of 7.7 Å) and a hexagonal cage (cage A, diameter of 30 Å, Figure 1). Desolvated MFM‐422 shows a BET surface area of 3296 m^2^ g^−1^ and a high thermal stability up to 500 °C (Figures S43–S44).

**Figure 2:**
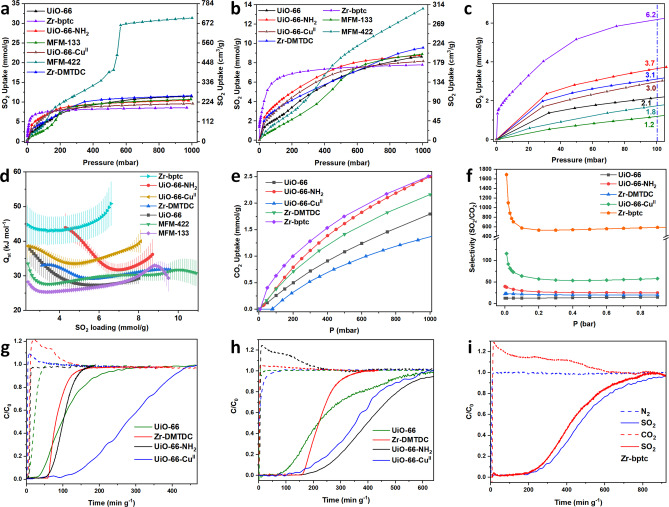
Gas adsorption, thermodynamic, selectivity and separation data. SO_2_ adsorption isotherms at (**a**) 273 K and 1 bar and (**b**) 298 K and 1 bar (desorption data are omitted for clarity and shown in Figures S1–S7); (**c**) SO_2_ adsorption isotherms from 0 to 0.1 bar at 298 K; (**d**) variation of *Q*_st_; (**e**) CO_2_ adsorption isotherms for Zr‐bptc, Zr‐DMTDC and UiO‐66 materials at 298 K and 1 bar; (**f**) comparison of IAST selectivities of SO_2_/CO_2_ (1 : 99) for Zr‐bptc, Zr‐DMTDC and UiO‐66 materials at 298 K; (**g**) breakthrough plots for a SO_2_/CO_2_ mixture (2500 ppm SO_2_, 15 % CO_2_ in He, total flow rate: 20 mL min^−1^) in Zr‐DMTDC and UiO‐66 materials at 298 K (solid line: SO_2_; dashed line: CO_2_); (**h**) breakthrough plots for a SO_2_/N_2_ mixture (2500 ppm SO_2_, 75 % N_2_ in He, total flow rate: 14 mL min^−1^) in Zr‐DMTDC and UiO‐66 materials at 298 K (solid line: SO_2_; dashed line: N_2_); (**i**) breakthrough plots for a SO_2_/N_2_ mixture (2500 ppm SO_2_, 75 % N_2_ in He, total flow rate: 40 mL min^−1^) and (2500 ppm SO_2_, 15 % CO_2_ in He, total flow rate: 40 mL min^−1^) in Zr‐bptc at 298 K (blue: SO_2_/N_2_ mixture; red: SO_2_/CO_2_ mixture).

Gravimetric adsorption isotherms of SO_2_ have been recorded for these MOFs at 273–298 K and from 0–1 bar (Figures 2a, b, S1–S7 and Table 1). MFM‐422 shows a SO_2_ uptake of 31.3 mmol g^−1^ at 273 K and 1.0 bar, comparable to the record previously achieved by UR3‐MIL‐101(Cr) (36.7 mmol g^−1^) under the same conditions.^[34]^ At 298 K and 1 bar, all 7 MOFs, i.e., UiO‐66, UiO‐66‐NH_2_, UiO‐66‐Cu^II^, Zr‐DMTDC, Zr‐bptc, MFM‐133 and MFM‐422, show fully reversible uptakes of SO_2_ of 8.6, 8.8, 8.2, 9.6, 7.8, 8.9 and 13.6 mmol g^−1^, respectively (Figures 2b and S1–S7). The multiple cycles of adsorption‐desorption of SO_2_ for all samples at 298 K show little change in the capacity, demonstrating excellent stability towards dry SO_2_ (Figures S1–S7, S30–S35). The comparable adsorption uptakes of UiO‐66, UiO‐66‐NH_2_ and UiO‐66‐Cu^II^ at 1 bar (8.2–8.8 mmol g^−1^) suggest that decoration of the pore environment with functional groups or open Cu^II^ sites has little impact on the total uptake capacity, which is determined primarily by the surface area. The slightly higher uptake of Zr‐DMTDC (9.6 mmol g^−1^) is consistent with its higher surface area (1345 m^2^ g^−1^), compared with the other three UiO‐66 materials. In contrast, enhancements in the uptake at 0.1 bar were observed for UiO‐66‐NH_2_, UiO‐66‐Cu^II^ and Zr‐DMTDC, compared with UiO‐66 (uptakes of 3.7, 3.0, 3.1 and 2.1 mmol g^−1^, respectively, Figure 2c). This demonstrates that the introduction of accessible −NH_2_, Cu^II^ or R‐S‐R sites into the pores can increase the binding strength with SO_2_ molecules. Interestingly, Zr‐bptc displays an extremely high uptake of 6.2 mmol g^−1^ at 0.1 bar and 298 K, suggesting potential for selective adsorption of SO_2_ at low concentration. The isosteric heats of adsorption (*Q*_st_) for SO_2_ uptake show decreasing values of 45–50, 44–32, 38–34, 32–29, 37–27, 31–27 and 26–19 kJ mol^−1^ for Zr‐bptc, UiO‐66‐NH_2_, UiO‐66‐Cu^II^, Zr‐DMTDC, UiO‐66, MFM‐422 and MFM‐133, respectively. Compared with UiO‐66, the materials UiO‐66‐NH_2_, UiO‐66‐Cu^II^ and Zr‐DMTDC show higher values for *Q*_st_, consistent with the enhanced adsorption at low pressure. The relatively low values of *Q*_st_ for MFM‐133 and MFM‐422 are consistent with their large pores, reducing the strength of host–guest interactions.

**Table 1:** Summary of BET surface areas, SO_2_ uptakes and *Q*_st_ and IAST selectivities in Zr‐MOFs.

MOFs	BET [m^2^ g^−1^]	SO_2_ Uptake [mmol g^−1^] at 1 bar		SO_2_ *Q*_st_ [kJ mol^−1^]	Selectivity	
		298 K	273 K		SO_2_/CO_2_ (1/99)	SO_2_/N_2_ (1/99)
Zr‐bptc	960	7.8	8.6	45–50	600	>5 000
UiO‐66‐Cu^II^	1068	8.2	9.6	38–34	54	3 100
UiO‐66‐NH_2_	1037	8.8	10.5	44–32	25	486
Zr‐DMTDC	1345	9.6	11.6	32–29	20	280
UiO‐66	1221	8.6	11.5	37–27	13	208
MFM‐133	2156	8.9	10.7	31–27	–	–
MFM‐422	3296	13.6	31.3	26–19	–	–

Adsorption isotherms of CO_2_ and N_2_ have also been recorded for Zr‐bptc, UiO‐66‐NH_2_, UiO‐66‐Cu^II^, Zr‐DMTDC and UiO‐66 to assess the adsorption selectivity (Figures 2e, S8–S12, Table S1). At 298 K, Zr‐bptc displays CO_2_ uptakes of 2.5 and 0.82 mmol g^−1^ at 1.0 and 0.15 bar, respectively. While UiO‐66‐NH_2_ and Zr‐ DMTDC display 58 % and 42 % enhancements in the CO_2_ uptake at 0.15 bar and 298 K compared with UiO‐66, UiO‐66‐Cu^II^ shows a reduction of CO_2_ uptake of 47 % at 0.15 bar and 298 K (Figures 2e). Thus, the latter has great potential for selective adsorption of SO_2_. Analysis of pure‐component isotherms via ideal adsorbed solution theory (IAST)^[35]^ affords adsorption selectivities for mixtures of SO_2_/CO_2_ (1/99) and SO_2_/N_2_ (1/99) (Figure 2f and S13) for Zr‐bptc, UiO‐66‐NH_2_, UiO‐66‐Cu^II^, Zr‐DMTDC and UiO‐66. Zr‐bptc displays high selectivities of 600 for SO_2_/CO_2_ and >5000 for SO_2_/N_2_; the very high IAST selectivity is subject to uncertainties owing to the extremely low adsorption of N_2_. UiO‐66‐Cu^II^, UiO‐66‐NH_2_, Zr‐DMTDC and UiO‐66 display IAST selectivities for SO_2_/CO_2_ of 54, 25, 20 and 13, and for SO_2_/N_2_ of 3100, 486, 280 and 208, respectively. To confirm the selective capture of SO_2_ under realistic concentrations,^[36]^ fixed‐beds packed with these MOFs were studied by dynamic breakthrough experiments with a mixture of SO_2_/CO_2_ (2500 ppm SO_2_/15 % CO_2_ in He) at 298 K and 1.0 bar. UiO‐66, UiO‐66‐NH_2_, Zr‐DMTDC and UiO‐66‐Cu^II^ exhibit retention times for SO_2_ in the expected order of 33, 53, 58 and 100 min g^−1^, respectively (Figure 2g). The same sequence was observed in the separation of the mixture of SO_2_/N_2_ (2500 ppm/75 %) with retention times of 80, 175, 157 and 175 min g^−1^ for UiO‐66, UiO‐66‐NH_2_, Zr‐DMTDC and UiO‐66‐Cu^II^, respectively (Figure 2h). Zr‐bptc shows highly selective retention of SO_2_ at 213 and 235 min g^−1^ for mixtures of SO_2_/CO_2_ (2500 ppm SO_2_/15 % CO_2_ in He) and SO_2_/N_2_ (2500 ppm SO_2_/75 % CO_2_ in He), respectively (Figure 2i). Thus, the breakthrough results are fully consistent with the isotherm data and confirm the positive role of open Cu^II^ sites on selective SO_2_ adsorption.

**Figure 3:**
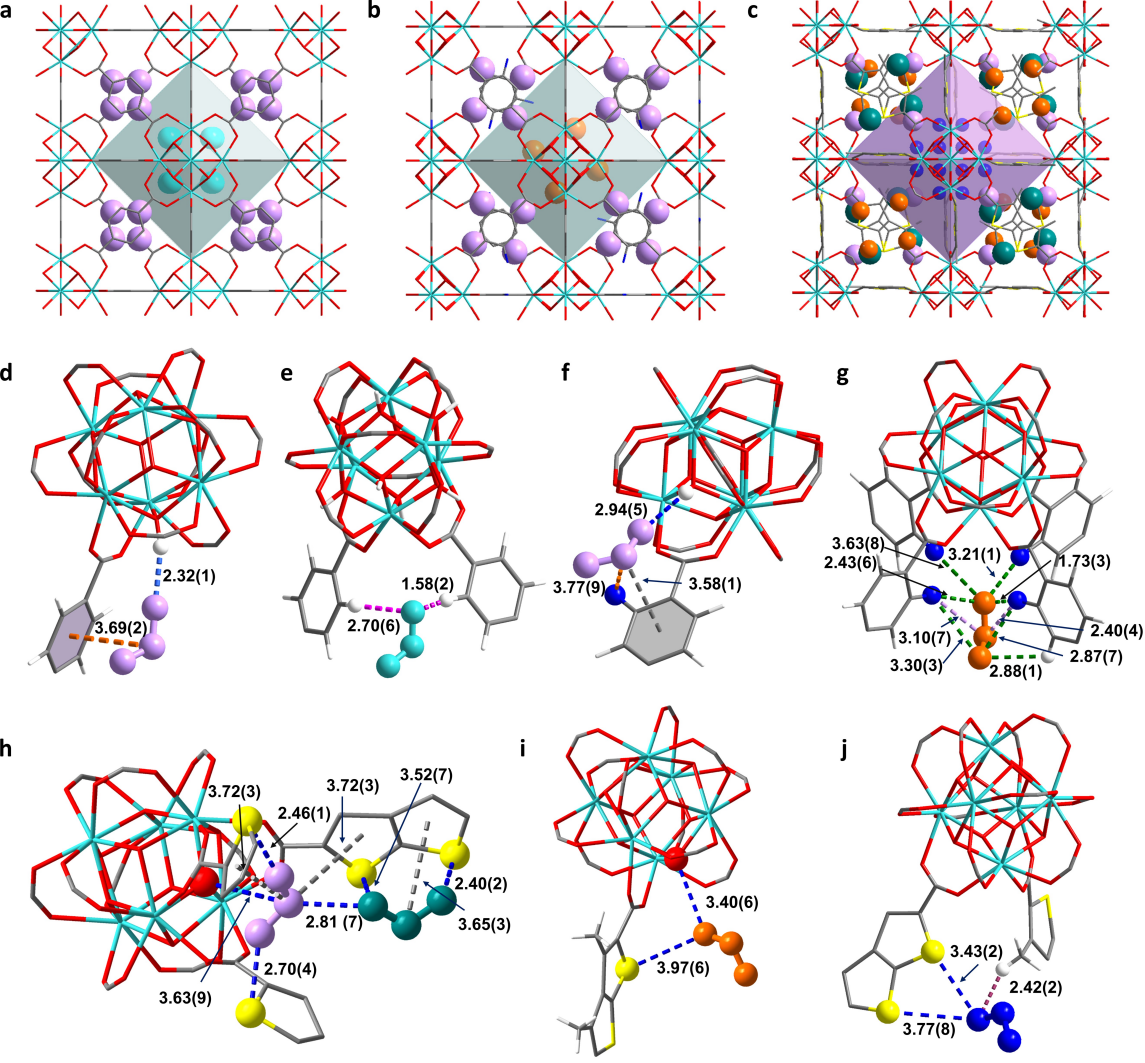
Views of binding of SO_2_ in (**a**) UiO‐66 (site I in cage T: lavender; site II in cage O: cyan); (**b**) UiO‐66‐NH_2_ (site I’ in cage T: lavender; site II’ in cage O: orange); (**c**) Zr‐DMTDC (site I’’, II’’ and III’’ in cage T: teal, lavender and orange, respectively; site IV’ in cage O: blue); (**d**) UiO‐66 at site I (SO_2_: lavender); (**e**) UiO‐66 at site II (SO_2_: cyan); (**f**) UiO‐66‐NH_2_ at site I’ (SO_2_: lavender); (**g**) UiO‐66‐NH_2_ at site II’ (SO_2_: orange); (**h**) Zr‐DMTDC at site I’′ (SO_2_: teal) and II’’ (SO_2_: lavender); (**i**) Zr‐DMTDC at site III’’ (SO_2_: orange); (**j**) Zr‐DMTDC at site IV’’ (SO_2_: blue) (in framework Zr: cyan; O: red; S: yellow; C: dark grey and H: white). All units are quoted in Å.

Rietveld refinements of the high‐resolution SXPD data of SO_2_‐loaded UiO‐66 [Zr_6_O_4_(OH)_4_(bdc)_6_ ⋅ (SO_2_)_7.7_] reveal two binding sites I and II located in cage T (SO_2_/{Zr_6_}=5.1) and cage O (SO_2_/{Zr_6_}=2.6), respectively (Figure 3a). The hydrogen bond [OSO⋅⋅⋅μ_3_‐HO=2.32(1) Å] and dipole–dipole interaction [O_2_ S⋅⋅⋅phenyl ring=3.69(2) Å] stabilise SO_2_ (I) (Figure 3d). SO_2_ (II) is stabilised by two hydrogen bonds [OSO⋅⋅⋅H−C=1.58(2), 2.70(6) Å] (Figure 3e). In SO_2_‐loaded UiO‐66‐NH_2_ [Zr_6_O_4_(OH)_4_(bdc−NH_2_)_6_ ⋅ (SO_2_)_8.1_], two binding sites I′ and II′ are observed in cage T (SO_2_/{Zr_6_}=4.7) and cage O (SO_2_/{Zr_6_}=3.4), respectively (Figure 3b). Due to the presence of active −NH_2_ groups, the adsorbed SO_2_ molecules are stabilised strongly by the formation of supramolecular interactions between −NH_2_ groups and SO_2_ molecules. A dipole–dipole interaction [NH_2_⋅⋅⋅SO_2_=3.77(9) Å] was identified and works together with an interaction [O_2_ S⋅⋅⋅phenyl ring=3.58(1) Å] and hydrogen bonding [OSO⋅⋅⋅μ_3_‐HO=2.94(5) Å] that stabilise SO_2_ binding at site I′ (Figure 3f). In addition, seven hydrogen bonds were identified [OSO⋅⋅⋅H−C=2.88(1) Å, SO_2_⋅⋅⋅NH_2_=1.73(3), 2.43(6), 2.87(7), 3.21(1), 3.30(3) and 3.63(8) Å], which work together with two further dipole–dipole interactions [O_2_ S⋅⋅⋅NH_2_=2.40(4) and 3.10(7) Å] to stabilise SO_2_ at site II′ (Figure 3g). The additional hydrogen bonds and dipole–dipole interactions demonstrate enhanced binding of SO_2_ in UiO‐66‐NH_2_, consistent with the increased SO_2_ adsorption at low pressure.

In SO_2_‐loaded Zr‐DMTDC [Zr_6_O_4_(OH)_4_(DMTDC)_2_ ⋅ (SO_2_)_13.1_], four binding sites were revealed (I′′‐IV′′). Sites I′′, II′′ and III′′ are localised in cage T (SO_2_/{Zr_6_}=4.2, 4.1 and 2.5, respectively) (Figure 3c). The SO_2_ molecule at site I′′ is stabilised by the formation of three dipole–dipole interactions [OSO⋅⋅⋅S‐ring=2.40(2) and 3.52(7) Å; O_2_ S⋅⋅⋅thiophene ring=3.65(3) Å] (Figure 3h). The SO_2_ molecules at site II′′ are stabilised further by four dipole–dipole interactions [OSO⋅⋅⋅S‐ring=2.46(1) and 2.70(4) Å; O_2_ S⋅⋅⋅thiophene ring=3.72(3) and 3.72(3) Å] and supramolecular interaction [O_2_ S⋅⋅⋅μ_3_‐O=3.63(9) Å]. In addition, dipole–dipole interaction between SO_2_ at sites I′′ and II′′ [OSO(I′′)⋅⋅⋅SO_2_(II′′)=2.81(7) Å] was identified (Figure 3h), which is not observed in either UiO‐66 or UiO‐66‐NH_2_ and may result from the slightly enlarged pore size. The formation of dipole–dipole interactions [O_2_ S⋅⋅⋅μ_3_‐O=3.40(6) Å] and [OSO⋅⋅⋅S‐ring=3.97(6) Å] were identified between SO_2_(III′′) and the framework (Figure 3i). Site IV′′ is in the cage O and stabilised by two dipole–dipole interactions [OSO⋅⋅⋅S‐ring=3.43(2) and 3.77(8) Å] and a hydrogen bond [OSO⋅⋅⋅H_3_C=2.42(2) Å] (Figure 3j). This crystallographic study enables direct observation of host–guest interactions, and revealed that the introduction of heteroatom S dominated the supramolecular interactions facilitating the immobilisation of SO_2_ at low pressure.

**Figure 4:**
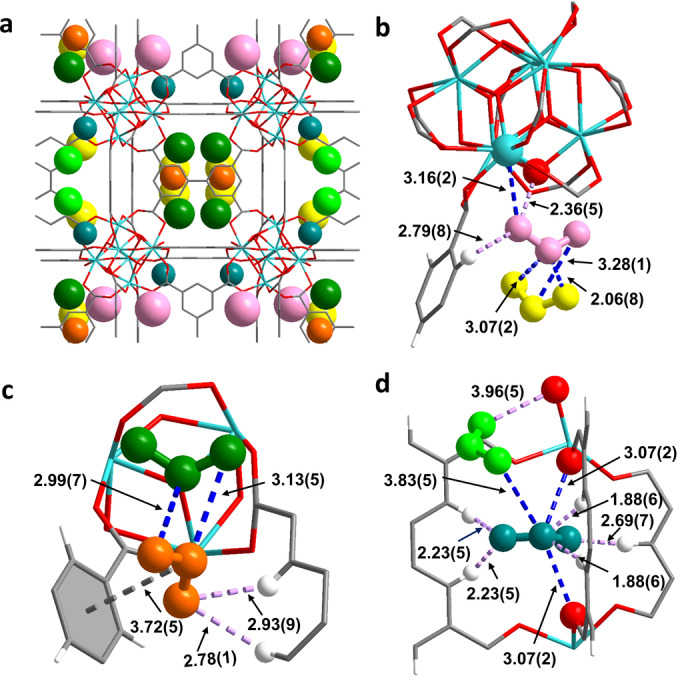
Views of the binding sites of SO_2_ in (**a**) Zr‐bptc (site I, II, III, IV and V in cage A: rose, yellow, dark green, green and orange, respectively; site VI in cage B: teal); (**b**) Zr‐bptc at sites I (SO_2_: rose) and II (SO_2_: yellow); (**c**) Zr‐bptc at site III (SO_2_: dark green) and V (SO_2_: orange); (**d**) Zr‐bptc at site IV (SO_2_: green) and VI (SO_2_: teal) (in framework Zr: cyan; O: red; S: yellow; C: dark grey and H: white). All units are quoted in Å.

In SO_2_‐loaded Zr‐bptc, [Zr_6_O_4_(OH)_4_(bptc)_3_ ⋅ (SO_2_)_5.8_], six binding sites were revealed (I–VI) (Figure 4a). Sites I, II, III, IV and V are localised in cage A with SO_2_/{Zr_6_} ratios of 1.7, 1.1, 1.0, 0.61 and 0.96, respectively, and Site VI located at cage B with a SO_2_/{Zr_6_} ratio of 0.43. SO_2_ molecules at site I were stabilised by Zr^IV^ sites [OSO⋅⋅⋅Zr=3.16(2) Å] and by two hydrogen bonds [OSO⋅⋅⋅H−C=2.79(8) Å and OSO⋅⋅⋅μ_3_‐OH=2.36(5) Å] (Figure 4b). SO_2_ molecules at site II are immobilised by three dipole–dipole interactions [OSO(II)⋅⋅⋅SO_2_(I)=2.06(8) and 3.07(2) Å, OSO(I)⋅⋅⋅SO_2_(II)=3.28(1) Å] with SO_2_ at site I (Figure 4b). SO_2_ molecules at site III are stabilised by dipole–dipole interactions [O_2_ S(III)⋅⋅⋅OSO(V)=2.99(7) Å, OSO(III)⋅⋅⋅SO_2_(V)=3.13(5) Å] with SO_2_ at site V immobilised by dipole–dipole interactions [O_2_ S⋅⋅⋅phenyl ring=3.72(5) Å] and two‐fold electrostatic interactions [OSO⋅⋅⋅H−C=2.78(1) and 2.93(9) Å] (Figure 4c). SO_2_ molecules at site IV are stabilised by a weak hydrogen bond [OSO⋅⋅⋅μ_3_‐HO=3.96(5) Å] and dipole–dipole interaction [OSO(IV)⋅⋅⋅SO_2_(VI)=3.83(5) Å] with SO_2_ at site VI (Figure 4d). SO_2_ molecules at site VI sit at the centre of cage B and are immobilised by five hydrogen bonds [OSO⋅⋅⋅H−C=1.88(6), 1.88(6), 2.23(5), 2.23(5) and 2.69(7) Å] and two dipole–dipole interactions [O_2_ S⋅⋅⋅OOC=3.07(2) and 3.07(2) Å] (Figure 4d). In contrast to the host–guest binding observed in SO_2_‐loaded UiO‐66 type systems, Zr^IV^ sites, the strong hydrogen bonding at site I, unique dipole–dipole interactions between SO_2_(VI) and carboxylic groups and multiple strong hydrogen bonding at site VI jointly facilitate the exceptional SO_2_ uptake at low pressure.

**Figure 5:**
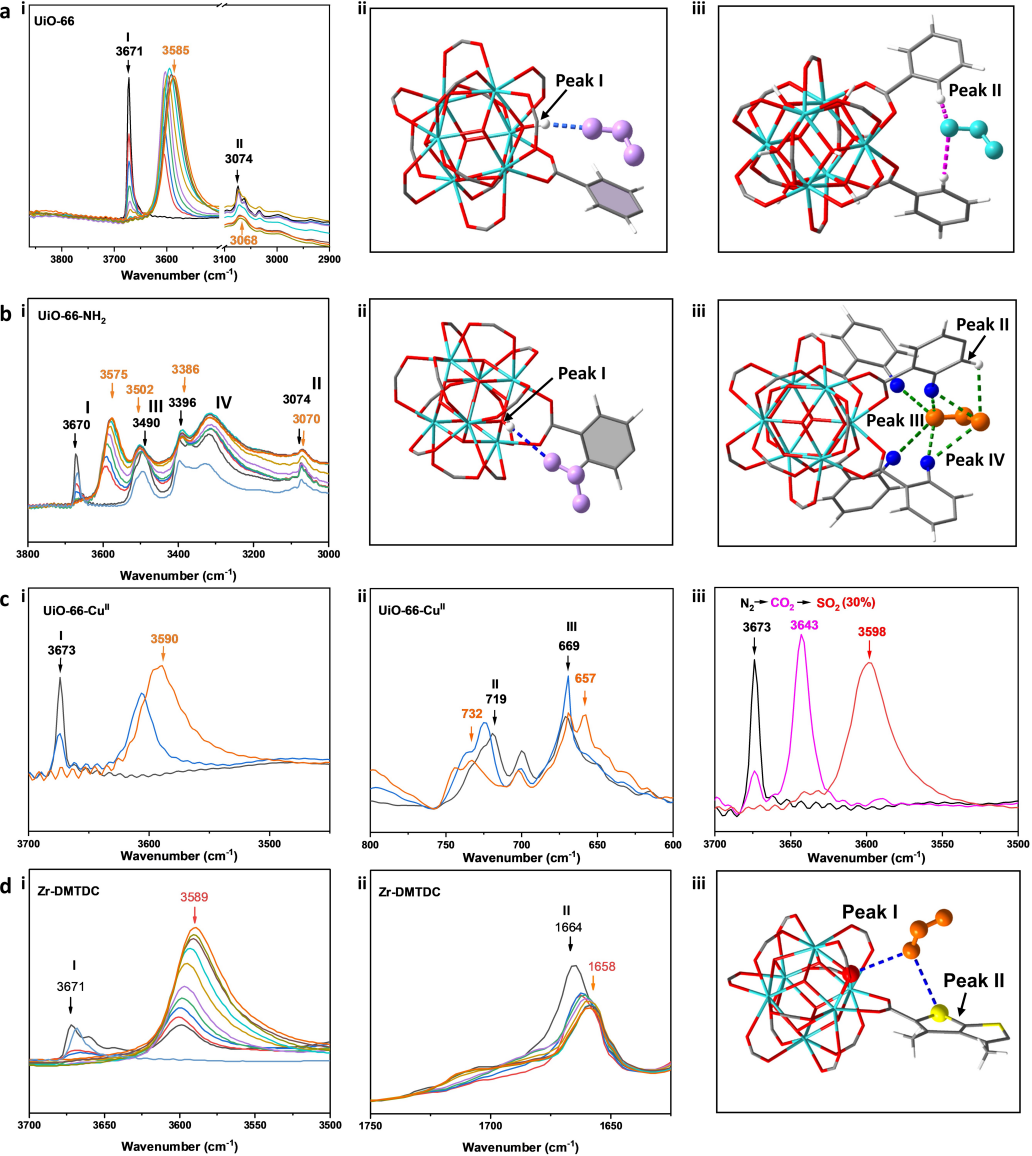
(**a**) (i). IR spectra showing the *ν*(*μ*_3_‐OH) and *ν*(*‐*CH) stretching region for UiO‐66 at various loadings of SO_2_; (ii–iii) views of corresponding structures. (**b**) (i) IR spectra showing the *ν*(*μ*_3_‐OH), *ν*(*‐*NH_2_) and *ν*(*‐*CH) stretching region for UiO‐66‐NH_2_ at various loadings of SO_2_; (ii‐iii) views of corresponding structures; (**c**) IR spectra of (i) *ν*(*μ*_3_‐OH), (ii) *ν*(Cu−OH) and *ν*(Cu−OH_2_) stretching region for UiO‐66‐Cu^II^ at 2 % (blue) and 100 % (orange) loading of SO_2_ (other loadings are omitted for clarity and shown in the Supplementary Information Figures S21–S22); iii) IR spectra of the *ν*(*μ*_3_‐OH) stretch region for bare (black), 100% CO_2_‐loading (purple) and 30% SO_2_‐loading for CO_2_ displacement (red) in UiO‐66‐Cu^II^; (**d**) IR spectra of (i) *ν*(*μ*_3_‐OH) and (ii) *ν*(S−C) stretching region for Zr‐DMTDC at various loadings of SO_2_; (iii). Views of corresponding structures (in various SO_2_‐loading experiments: black: bare MOF, red: 1% SO_2_‐loading, blue: 2% SO_2_‐loading, green: 5% SO_2_‐loading, violet: 10% SO_2_‐loading, dark yellow: 20% SO_2_‐loading, cyan: 40% SO_2_‐loading, light wine: 60% SO_2_‐loading, wine: 80% SO_2_‐loading, orange: 100% SO_2_‐loading).

The binding dynamics of adsorption of SO_2_ (0–1 bar) in the UiO‐66 type systems have been analysed by in situ synchrotron infrared micro‐spectroscopy. For all the MOFs, clear binding of SO_2_ to the hydroxyl group is observed with a red shift of the −OH stretching vibration at ≈3671 cm^−1^ by 86, 95, 83 and 82 cm^−1^ in UiO‐66, UiO‐66‐NH_2_, UiO‐66‐Cu^II^ and Zr‐DMTDC, respectively (Figures 5ai–di). There is clear evidence for enhanced interaction of SO_2_ via hydrogen bonding to −NH_2_ groups ([NH_2_⋅⋅⋅OSO], the characteristic NH_2_ band shifting from 3490 to 3502 cm^−1^ and 3396 to 3386 cm^−1^ (Figure 5b). Dipole interactions are observed to the Cu^II^ sites ([OSO⋅⋅⋅⋅Cu−OH], with the characteristic Cu‐OH band shifting from 719 to 732 cm^−1^. Formation of [OSO⋅⋅⋅Cu−OH_2_] interactions leads to a new band at 657 cm^−1^ assigned to the CuO stretching vibration^[37]^ (Figure 5cii), while the thiophene system leads to [OSO⋅⋅⋅S] interactions with a characteristic shift in the S−C stretch from 1664 to 1658 cm^−1[38]^ (Figure 5dii). The displacement and cooperative binding of SO_2_ and CO_2_ was investigated in UiO‐66‐Cu^II^. The *ν*(*μ*_3_‐OH) mode was monitored to examine the displacement of bound CO_2_ by SO_2_ (Figures 5ciii, S26). Upon loading of CO_2_ to 1 bar, the peak areas for the *ν*(*μ*_3_‐OH) stretch corresponding to the bare and CO_2_‐loaded materials are approximately equal. Due to weak interaction between CO_2_ and the *μ*_3_‐OH group, the bare *μ*_3_‐OH band is not fully depleted but a new peak at 3643 cm^−1^ appears and is assigned to the [OH⋅⋅⋅OCO] band (Figure 5ciii).

Upon stepwise dosing of the CO_2_‐saturated material with SO_2_ (i.e., tuning the SO_2_/CO_2_ mixture from 0/100 to 100/0 while maintaining a total pressure of 1.0 bar), there is a steady change in the *ν*(*μ*_3_‐OH) region that includes new bands appearing in a similar manner to the pure SO_2_ experiment, indicating that bound CO_2_ does not impede SO_2_ adsorption (Figure S26). Upon 30 % SO_2_‐loading, the characteristic [OH⋅⋅⋅OCO] band has fully disappeared showing that SO_2_ readily displaces bound CO_2_ in the pore as a result of stronger binding. Hence, selective capture of SO_2_ from a mixture of SO_2_/CO_2_ can be achieved as demonstrated in separation experiments. Furthermore, 40 %, 45 % and 50 % SO_2_‐loadings fully displace CO_2_ in UiO‐66‐NH_2_, Zr‐DMTDC and UiO‐66, respectively (Figures S24–27). The competitive binding studies of SO_2_/CO_2_ further confirm enhanced SO_2_ binding in the decorated MOFs. The decreasing partial pressure of SO_2_ on full displacement of CO_2_ in UiO‐66‐Cu^II^, UiO‐66‐NH_2_ and Zr‐DMTDC is consistent with that observed in static and dynamic adsorption studies.

**Figure 6:**
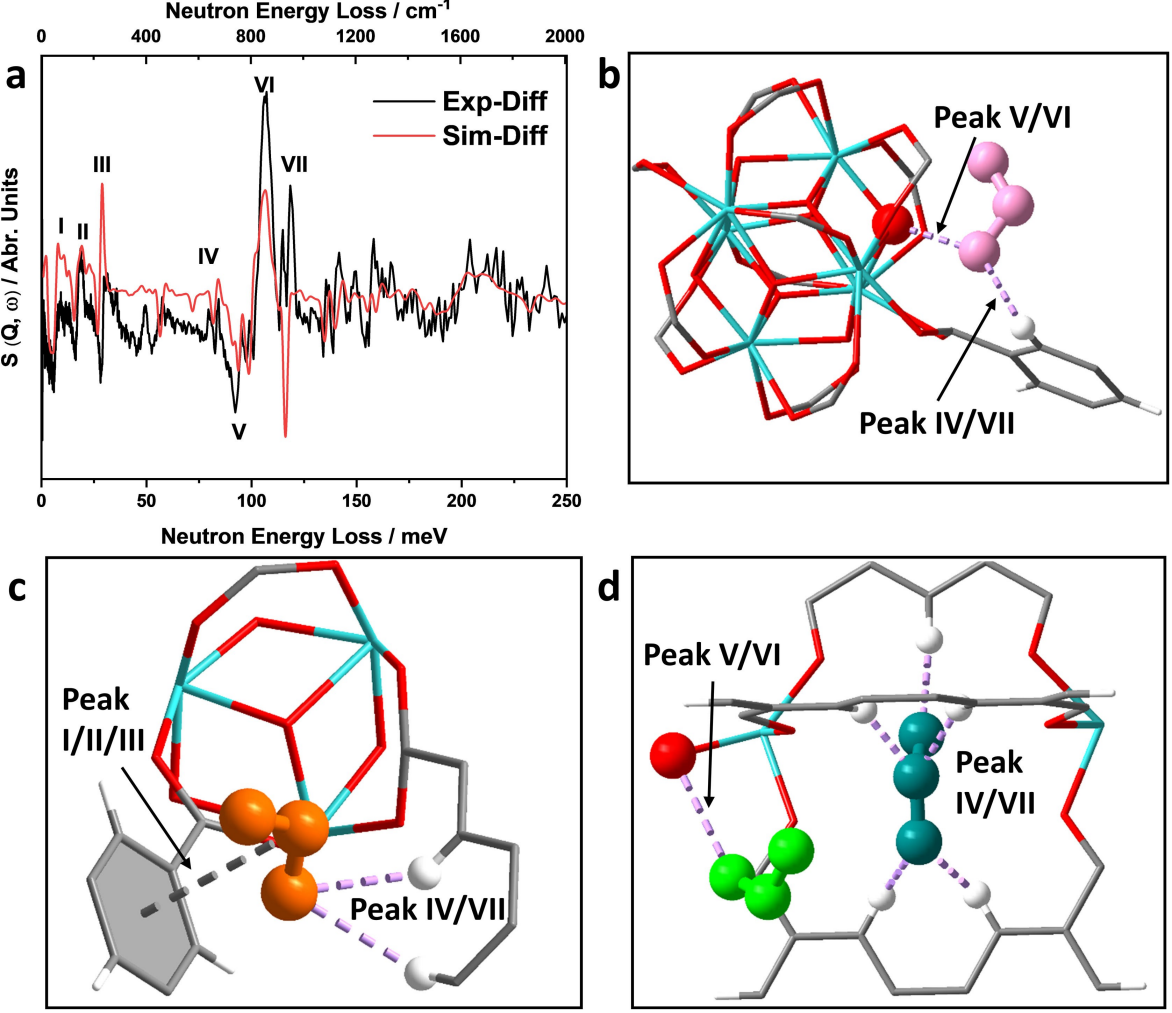
(**a**) Comparison of the difference plots for experimental and DFT‐calculated INS spectra of bare and SO_2_‐loaded Zr‐bptc. No scale factor was used for the DFT calculations. *S*, dynamic structure factor; *Q*, difference between incoming and outgoing wave vector; ω, the energy change experienced by the sample; (**b**–**d**) Views of corresponding structures.

*In situ* INS, coupled with DFT calculations, enables the visualization of binding dynamics for SO_2_‐loaded Zr‐bptc. Seven major changes in the INS spectra were observed on the adsorption of SO_2_ in Zr‐bptc (Figure 6a). Peaks I‐III occur at low energy transfer (<60 meV) and Peaks IV‐VII at the high energy region (80–120 meV). Peak I (8.3 meV) is assigned to the flapping mode of the aromatic ring and peaks II (19.3 meV) and III (29.6 meV) are due to aromatic deformation. Peaks IV (83.7 meV) and VII (118 meV) are assigned to C−H out‐of‐plane bending modes with H moving in the same direction and opposite directions, respectively. The changes in peaks I, II, III, IV and VII suggest interactions between adsorbed SO_2_ molecules and the aromatic moieties, consistent with the crystallographic analyses (Figures 6b–d). Peaks V (92.9 meV) and VI (106.5 meV) are assigned to μ_3_‐OH wagging and twisting, respectively, and their changes support the formation of hydrogen bonds [OSO⋅⋅⋅μ_3_‐HO] that were observed in the crystallographic analysis (Figure 6b and 6d).

**Scheme 1:**
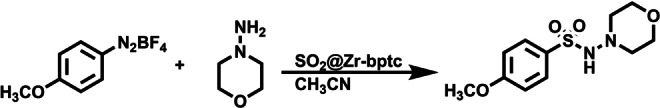
Conversion of captured SO_2_ in Zr‐bptc for the synthesis of fine chemicals; 1 equivalent of 4‐methoxy‐aryldiazonium tetrafluoroborate and 5 equivalents of amine and SO_2_‐loaded Zr‐bptc were reacted at room temperature for 1 h.

Unlike FGD technology, where SO_2_ is bound permanently to sorbent materials to form solid inorganic wastes, the SO_2_ captured by these Zr‐MOFs remains available to undergo chemical transformation to valuable products. Here, a proof‐of‐concept experiment on aminosulfonylation[^39^, ^40^] using the SO_2_‐loaded Zr‐bptc was conducted, and quantitative conversion of the captured SO_2_ was achieved to give 4‐methoxyl‐aryldiazonium tetrafluoroborate in 85 % yield (Scheme 1). Upon regeneration, Zr‐bptc can be used for at least 3 cycles without any change in the crystal structure or porosity of the material (Figure S46 and Table S5), thus demonstrating its great potential of the capture and conversion of waste SO_2_ to fine chemicals.

## Conclusion

Powerful drivers exist for the development of new regenerable sorbents for SO_2_ to enable its recovery from exhaust gases and conversion into chemical feedstocks. The highly corrosive and reactive nature of SO_2_ leads generally to severe structural degradation of sorbent materials. We report the positive impacts on low pressure SO_2_ uptake by introducing functional groups and atomically‐dispersed Cu^II^ sites into a family of Zr‐MOFs. Owing to the confined metal–ligand cages in Zr‐bptc, an exceptional uptake of SO_2_ (6.2 mmol g^−1^) was observed at 0.1 bar and 298 K. Furthermore, the captured SO_2_ in Zr‐bptc can be converted readily into fine chemicals, paving new pathways to “waste‐to‐chemicals” technologies. In situ SXPD, microFTIR and INS studies, coupled with DFT calculations, unravel the molecular details of host–guest binding that result in the enhancement of SO_2_ adsorption at low pressure in these materials. These studies confirm that control of pore environments is an important approach for improving the adsorption of SO_2_.

## Associated Content

Additional crystallographic information, gas adsorption data, thermogravimetric analysis, density function theory (DFT) calculations and breakthrough data are available in the Supporting Information. The crystal structures of [Zr_6_(OH)_8_(OH)_8_(tcpt)_2_], [Zr_6_O_4_(OH)_4_(bptc)_3_ ⋅ (SO_2_)_5.8_], [Zr_6_O_4_(OH)_4_(DMTDC)_6_ ⋅ (SO_2_)_13.1_], [Zr_6_O_4_(OH)_4_(bdc)_6_ ⋅ (SO_2_)_7.7_] and [Zr_6_O_4_(OH)_4_(bdc−NH_2_)_6_ ⋅ (SO_2_)_8.1_] are available free of charge from the Cambridge Crystallographic Data Centre (Deposition Numbers 2132832, 2151090, 2151089, 2151088 and 2151087).

## Conflict of interest

The authors declare no conflict of interest.

1

## Supporting information

As a service to our authors and readers, this journal provides supporting information supplied by the authors. Such materials are peer reviewed and may be re‐organized for online delivery, but are not copy‐edited or typeset. Technical support issues arising from supporting information (other than missing files) should be addressed to the authors.

Supporting Information

Supporting Information

Supporting Information

Supporting Information

Supporting Information

## Data Availability

The data that support the findings of this study are available in the supplementary material of this article.
