# Supplementary material for: Structural and Dynamic Analysis of Sulphur Dioxide Adsorption in a Series of Zirconium‐Based Metal–Organic Frameworks
Source: Angew Chem Int Ed Engl. 2022 Jul 25;61(36):e202207259. doi: 10.1002/anie.202207259 (PMC9546045; doi:10.1002/anie.202207259)
Supplement: Supplementary file 2 — Supporting Information [file ANIE-61-0-s005.pdf]

Angewandte Chemie
Eine Zeitschrift der Gesellschaft Deutscher Chemiker

Supporting Information

Structural and Dynamic Analysis of Sulphur Dioxide Adsorption in a Series of Zirconium-Based Metal–Organic Frameworks

J. Li, G. L. Smith, Y. Chen, Y. Ma, M. Kippax-Jones, M. Fan, W. Lu, M. D. Frogley, G. Cinque, S. J. Day, S. P. Thompson, Y. Cheng, L. L. Daemen, A. J. Ramirez-Cuesta, M. Schröder, S. Yang**

Supplementary Information

Structural and Dynamic Analysis of Adsorption of Sulphur Dioxide in a Series of Zirconium-based Metal-Organic Frameworks

Jiangnan Li[a], Gemma L. Smith[a], Yinlin Chen[a], Yujie Ma[a], Meredydd Kippax-Jones[a], [b], Mengtian Fan[a], Wanpeng Lu[a], Mark D. Frogley[b], Gianfelice Cinque[b], Sarah J. Day[b], Stephen P. Thompson[b], Yongqiang Cheng[c], Luke L. Daemen[c], Anibal J. Ramirez-Cuesta[c], Martin Schröder*[a] and Sihai Yang*[a]

[a] Department of Chemistry, University of Manchester, Manchester, M13 9PL (UK)

[b] Diamond Light Source, Harwell Science Campus, Oxfordshire, OX11 0DE (UK)

[c] Neutron Scattering Division, Neutron Sciences Directorate, Oak Ridge National Laboratory, Oak Ridge, TN 37831 (USA)

1. Experimental Section…………………………………………………………………… …....3
1.1 Synthesis and Activation of Zr-based MOFs……………………………………….………3
1.2 SO_2 Safety……………………………………………………………………………...4
1.3 Gas Adsorption Isotherms ………………………………………………………….……..4
1.4 Gas Separation by Breakthrough Experiments ………………………………………......5
1.5 *In situ* Synchrotron X-Ray Powder Diffraction………………………………………..5
1.6 Inelastic Neutron Scattering (INS)…………………………………………….……..5
1.7 DFT Modelling and Simulation……………………………………………………………6
1.8 *In situ* FT-IR Micro-spectroscopy…………………………………………………………6
1.9 Analysis of the IAST Selectivity …………………………………………………….…..6
1.10 Powder X-ray Diffraction (PXRD) and BET Surface Area Analysis……………….….......…….6
1.11 Thermo-gravimetric Analysis ……………………………………………………….....7
2. Adsorption Isotherms…………………………………...........................…………..7
3. Selectivity Data for Zr-MOFs……………………………………..……....................…....15
4. *In situ* Synchrotron X-Ray Powder Diffraction ……………………..……...............……...16
5. *In situ* Infrared Spectroscopy…………………………………..…….……......................20
6. *In situ* Inelastic Neutron Scattering ……....……………………………………...........................25
7. PXRD patterns for Zr-MOFs………………………………………………………..……..26
8. Calculation of Isosteric Heats of Adsorption…………………………………………….…..30
9. N_2 isotherms at 77 K of MFM-422………………………………………………….…..34
10. Thermo-gravimetric Analysis………………………………...........…....………………..35
11. Conversion of Captured SO_2……………………………………………………………...36
12. References……………………………………………………………………….……….....38

1. Experimental Section

1.1 Synthesis and Activation of MOFs

All reagents were used as received from commercial suppliers without purification. Synthesis and activation of UiO-66, UiO-66-NH_2, UiO-66-Cu^{II}, Zr-DMTDC, MFM-133 and Zr-bptc were carried out using the reported methods[1-6].

Synthesis of ligands

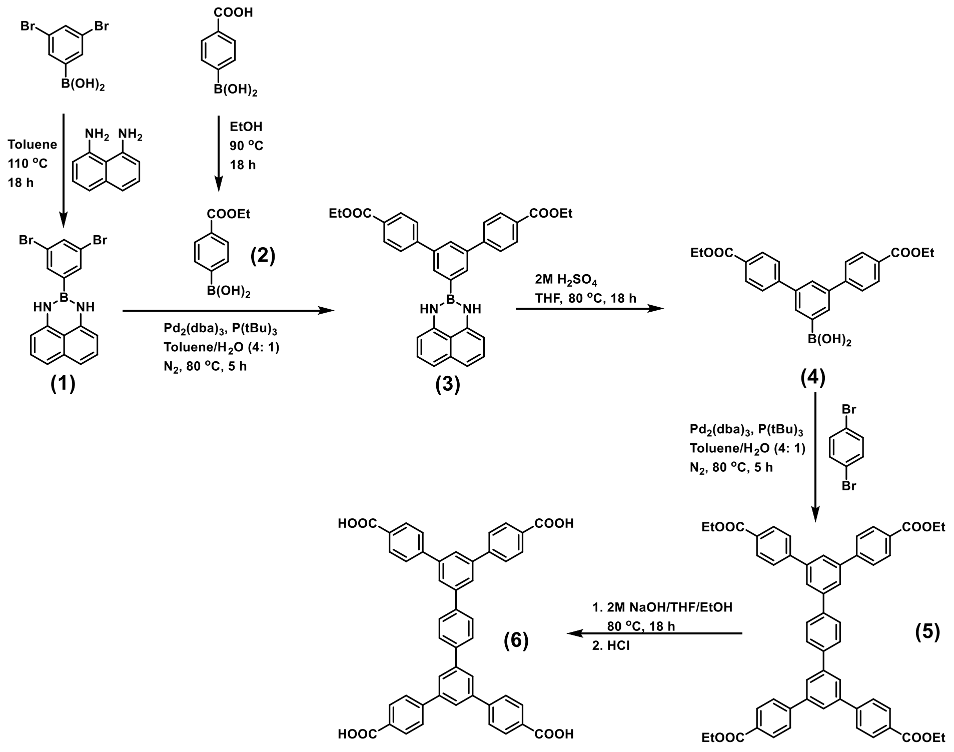

Synthesis of Compound (1)

3, 5-Dibromobenzene boronic acid (10.0 g, 0.036 mol) and 1, 8-diaminonaphthalene (6.8 g, 0.045 mol) was stirred in toluene (150 mL) at 110 °C for 4 h. The reaction mixture was cooled and the product purified through a silica plug and recrystallized from CH_2Cl_2/petroleum ether. The product was collected as bright yellow needles (12.6 g, 87%). ^{1}H NMR δ (400 MHz, $CDCl_3$): 7.75 (1H, t, J = 1.8 Hz), 7.66 (2H, d, J = 1.8 Hz), 7.18-7.05 (4H, m), 6.41 (2H dd, J = 7.2, 1.1 Hz). 5.92 (2H, br, s, NH); ^{13}C NMR δ (100 MHz; $CDCl_3$): 140.4, 136.3, 135.5, 133.0, 128.7, 123.5 ppm.

Synthesis of Compound (2)

To a stirred solution of 4-carboxybenzene boronic acid (20.0 g, 0.12 mol) in EtOH (500 mL) was added H_2SO_4 (6 mL). The reaction mixture was stirred at 90 °C for 18 h, after which it was cooled and reduced in volume *in vacuo* (*ca.* 200 mL). The white solid was crashed out of solution with the addition of water and was collected by filtration. The product was washed with excess water and dried in an oven (80 °C) overnight to yield a white solid (19.5 g, 84%). ^{1}H NMR δ (270 MHz, DMSO-d_6): 7.94-7.84 (4H, m, Ar), 4.29 (2H, q, J=7.1 Hz), 1.29 (3H, t, J=7.1 Hz); ^{13}C NMR δ (100 MHz; DMSO-d_6): 165.5, 144.2, 134.1, 131.5, 129.0, 61.1, 14.6 ppm.

Synthesis of Compound (3)

To a stirred solution of compound **2** (6.96 g, 35.9 mmol) and K_2CO_3 (4.34 g, 31.4 mmol) in water/toluene (*v/v* = 100 mL; 400 mL) was added compound **1** (5.15 g, 12.8 mmol). The reaction mixture was degassed at 60 °C for 1 h, after which was added tri-tert-butylphosphine (1 M in toluene, 3.8 mL) and $Pd_2(dba)_3$ (1.17 g, 1.3 mmol). The reaction mixture was stirred at 80 °C under an inert atmosphere for 5 h and then filtered whilst

hot. The product was extracted with CH_2Cl_2 and washed with water. The organic layer was separated, dried with $MgSO_4$ and the solvent removed under reduced pressure. Recrystallization of the crude product from CH_2Cl_2/MeOH afforded the product as a yellow solid (5.05 g, 73%). ^{1}H NMR δ (270 MHz, $CDCl_3$): 8.16 (4H, d, *J*=8.5 Hz, Ar), 7.90 (1H, t, *J*=1.8 Hz, Ar), 7.86 (2H, d, *J*=1.8, Ar), 7.76 (2H, d, J=1.9 Hz, Ar), 7.72 (2H, d, *J*=1.8, Ar), 7.20-7.04 (4H, m, Ar), 6.46 (2H, dd, *J*= 7.1, 1.2 Hz, Ar), 6.14 (2H, s), 4.41 (4 H, *J*=7.1), 1.42 (6H, t, *J*=7.1); ^{13}C NMR δ (68 MHz; $CDCl_3$): 166.6, 145.2, 140.9, 136.6, 130.3, 130.2, 129.8, 128.3, 127.8, 127.3, 118.2, 106.3, 61.3, 14.5 ppm.

Synthesis of Compound (4)

Compound **3** (4.85 g, 8.42 mmol) was stirred in H_2SO_4 (2 M, 60 mL) and THF (245 mL) at 80 °C for 18 h. The reaction mixture was reduced in volume *in vacuo* until white precipitate was seen and excess water (*ca.* 500 mL) was added. The solid was filtered and washed with copious water (3.35 g, 96%).^{1}H NMR δ (270 MHz, DMSO-d_6): 8.20 (2H, d, *J*=1.8 Hz), 8.11-7.90 (9H, m), 4.34 (4H, q *J*= 7.1 Hz). 1.34 (6H, t, *J*=7.1Hz). ^{13}C NMR δ (68 MHz, DMSO-d_6): 166.2, 145.3, 139.4, 137.5, 133.3, 130.3, 129.4, 127.8, 61.4, 14.8 ppm.

Synthesis of Compound (5)

Compound **4** (3.35 g, 8.72 mmol) was added to a stirred solution of dibromobenzene (0.68 g, 2.90 mmol) and K_2CO_3 (2.0 g) in water/toluene (*v/v* = 65/ 260 mL). The reaction mixture was degassed at 60 °C for 1 h, after which was added *tri*-tert-butylphosphine (1 M in toluene, 1.7 mL) and $Pd_2(dba)_3$ (0.53 g, 0.56 mmol). The reaction mixture was stirred at 80 °C under an inert atmosphere for 5 h and then filtered whilst hot. The product was extracted with CH_2Cl_2 and the solution washed with water. The organic layer was separated, dried with $MgSO_4$ and the solvent removed *in vacuo*. Recrystallization of the crude product from CH_2Cl_2/MeOH afforded the product as a white solid (1.61 g, 68%). ^{1}H NMR δ (270 MHz, $CDCl_3$): 8.17 (8H, d, *J*= 8.4, Ar H), 7.92-7.74 (18H, m, Ar H), 4.42 (8H, q, *J*= 7.1 Hz), 1.42 (12H, t, *J*=7.1 Hz) ppm; ^{13}C NMR δ (100 MHz, $CDCl_3$): 19.9, 58.5, 86.8, 96.6, 106.9, 125.8, 125.9, 127.2, 129.43, 130.2, 131.4, 179.0, 179.5 ppm.

Synthesis of Compound (6)

Compound **5** (0.80 mg, 0.97 mmol) was added to a stirred solution of NaOH (2 M, 100 mL) and EtOH/THF (*v/v* = 1:1; 200 mL) and refluxed at 90 °C for 5 h. The organic solvent was removed *in vacuo* and the aqueous portion was acidified with HCl (2 M) until a white precipitate was seen. The fine solid was filtered, washed with water and dried in an oven to give a black solid, which was recrystallized from DMF/water to give a grey solid (530 mg, 79%). ^{1}H NMR δ (400 MHz; DMSO-d_6): 13.08 (4H, s), 8.19-7.99 (26H, m, Ar) ppm. ^{13}C NMR δ (100 MHz, DMSO-d_6): 167.2, 144.6, 141.9, 141.2, 139.8, 130.4, 128.4, 128.0, 125.9, 125.5 ppm.

Synthesis of MFM-422

Compound **6** (16 mg, 23 μmol), $ZrCl_3$ (21 mg, 90 μmol) and benzoic acid (620 mg, 5.08 mmol) were added to diethylformamide (3 mL) and heated in a sealed pressure tube in an oil bath at 120 °C for 2 days. The reaction mixture was filtered whilst still warm (*ca.* 50 °C) and washed twice with hot DMF, acetone and briefly dried in air to yield the zirconium MOF, MFM-422 (as synthesised), as a white microcrystalline material (10 mg, 30%) $Zr_6(OH)_8(OH)_8(tcpt)_2$, [H_4tcpt = Compound **6**]. IR (ATR) cm^{-1}: 1595 (phenyl ring C = C), 1412 (O-H), 1016 (C-O) and 777(C-H); Elemental analysis (% calculated/found): C 52.7/52.1 H2.9/2.7.

1.2 SO_2 safety

The hardware and piping involved in the supply, delivery and measurement of SO_2 were rigorously leak tested and used only within range of a SO_2 detection system with a sensitivity of 0.1 ppm.

1.3 Gas Adsorption Isotherms

Gravimetric sorption isotherms of SO_2 were recorded on a Hiden Xemis system under ultra-high vacuum (10^{-10} bar) using a turbo pumping system at 273, 278, 293 and 298 K, the temperature being maintained by a temperature-programmed water bath. Ultra-pure research grade (99.999%) SO_2 was purchased from BOC. In a typical gas adsorption experiment, the acetone-exchanged MOF sample (50 mg) was loaded onto the Xemis system and activated at 393 or 573 K under dynamic high vacuum (10^{-10} bar measured at pump) for 24 h to

give fully desolvated MOF sample. Gravimetric sorption isotherms for N_2 and CO_2 were recorded on a Hiden Isochema IGA-003 system or a Hiden Xemis system under ultra-high vacuum (10^{-10} bar) using a turbo pumping system at 273, 283, 293, 298 K, the temperature being maintained by a temperature-programmed water bath. Ultra-pure research grade (99.999%) N_2 and CO_2 were purchased from BOC or Air Liquide. In a typical gas adsorption experiment, 50 mg of acetone-exchanged MOF sample was loaded onto the Xemis/IGA system and activated at 393 or 573 K under dynamic high vacuum (10^{-10} bar) for 24 h to give fully desolvated MOF sample.

1.4 Gas Separation by Breakthrough Experiments

Breakthrough experiments were performed on a Hiden Isochema IGA-003 with ABR attachments and a Hiden Analytical mass spectrometer to detect the gases as they break through the sample bed. Experiments were carried out in a 7 mm diameter fixed-bed of 120 mm length packed with MOF powder (particle size < 1 micron). The sample was pre-activated at 393 or 573 K under vacuum and the pre-activated sample was loaded to the column and re-activated under a flow of He for 12 h. The fixed-bed was cooled to room temperature (298 K) using a temperature programmed water bath and the breakthrough experiment performed with a series of gas mixtures at atmospheric pressure and room temperature. The flow rate of the entering gas mixture was maintained at 14-40 mL min^{-1}, and the gas concentration, C, of gases at the outlet determined by mass spectrometry and compared with the corresponding inlet concentration C_0, where C/C_0 =1 indicates complete breakthrough.
For UiO-66 sample: 0.5 g sample was loaded onto the sample bed.
For UiO-66-NH_2 sample: 0.38 g sample was loaded onto the sample bed.
For UiO-66-Cu^{II} sample: 0.37 g sample was loaded onto the sample bed.
For Zr-DMTDC sample: 0.5 g sample was loaded onto the sample bed.
For Zr-bptc sample: 0.55 g sample was loaded onto the sample bed.

1.5 *In situ* Synchrotron X-Ray Powder Diffraction

High-resolution X-ray powder diffraction of SO_2-loaded MOFs was carried out on beamline I11 of the Diamond Light Source. A high brightness monochromatic beam was produced by a Si(111) monochromator and double-bounce harmonic rejection mirrors. The beam was delivered to the main instrument hutch where five multi-analysing crystal-detectors (MAC) travel in an arc of 2θ around the sample. Measurements were carried out in capillary mode and the sample environment controlled using an Oxford Cryosystems open-flow N_2 gas cryostat. The samples were ground to provide a uniform particle size, packed into a borosilicate capillary. The sample was activated under vacuum (1 x 10^{-6} mbar) at 393 or 573 K for > 3 h to remove residual solvent molecules from the material. Diffraction data for the activated sample were collected and analysed to confirm that no residual solvent molecules are present in the pores. Wavelength and capillary details are tabulated in Tables S3.

1.6 Inelastic Neutron Scattering (INS)

INS spectra were recorded on the VISION spectrometer at Spallation Neutron Source, Oak Ridge National Laboratory (USA). VISION is an indirect geometry crystal analyser instrument that provides a wide dynamic range with high resolution. The sample of pre-activated Zr-bptc (573 K under vacuum) was loaded into a cylindrical vanadium sample container with an indium vacuum seal and connected to a gas handling system. The sample was degassed at 10^{-7} mbar at 393 K for 1 day to remove any remaining trace guest water molecules. The temperature during data collection was controlled using a closed cycle refrigerator (CCR) cryostat (10 ± 0.1 K). The loading of SO_2 was performed volumetrically at room temperature in order to ensure that SO_2 was present in the gas phase when not adsorbed and also to ensure sufficient mobility of SO_2 inside the crystalline structure of Zr-bptc. Subsequently, the temperature was reduced to below 10 K in order to perform the scattering measurements with minimum thermal motion for the framework host and adsorbed SO_2 molecules. Background spectra [sample can plus bare Zr-bptc] were subtracted to obtain the difference spectra.

1.7 DFT Modelling and Simulation

Vibrational frequencies and polarization vectors were calculated using CP2K (http://www.cp2k.org)[7], based on the mixed Gaussian and plane-wave scheme[8] and the Quickstep module[9]. The calculation used molecularly optimized Double-Zeta-Valence plus Polarization (DZVP) basis set[10], Goedecker-Teter-Hutter pseudopotentials[11], and the Perdew-Burke-Ernzerhof (PBE) exchange correlation functional[12]. The plane-wave energy cutoff was 400 Ry. The DFT-D3 level correction for dispersion interactions, as implemented by Grimme *et al*[13], was applied with a cut-off distance of 15 Å. The calculation was performed at the Gamma point only with no symmetry constraint. Structural optimization was performed using the Broyden-Fletcher-Goldfarb-Shannon (BFGS) optimizer, until the maximum force is below 0.00045 Ry/Bohr (0.011 eV/Å). Finite displacement method was used for the phonon calculation, with incremental displacement of 0.01 Bohr (0.0053 Å). The INS spectrum was then simulated using the OClimax software[14].

1.8 *In situ* FT-IR Micro-spectroscopy

Synchrotron infrared micro-spectroscopy experiments were carried out at the Multimode Infrared Imaging and Microspectroscopy (MIRIAM) beamline at the Diamond Light Source, UK. The instrument is comprised of a Bruker Hyperion 3000 microscope in transmission mode, with a 15× objective and condenser and liquid N_2 cooled MCT detector, coupled to a Bruker Vertex 80 V Fourier Transform IR spectrometer using radiation generated from a bending magnet source. Spectra were collected (256 scans) in the range 650-4000 cm^{-1} with 4 cm^{-1} resolution and an infrared spot size at the sample of about 25 × 25 μm.

Acetone-exchanged samples of UiO-66, UiO-66-NH_2, UiO-66-Cu^{II} and Zr-DMTDC were placed onto a ZnSe disk within a Linkam FTIR600 gas-tight sample cell equipped with ZnSe windows, a heating stage and gas inlet and outlets. The gases were dosed volumetrically into the sample cell using mass flow controllers, the total flow rate being maintained at 100 mL min^{-1} for all experiments. The gases were vented directly to an exhaust system and the total pressure in the cell was therefore 1 bar for all experiments. The sample was dehydrated under a flow of dry N_2 at 100 mL min^{-1} and 393 K for 5 h. The sample was cooled to 298 K under a continuous flow of N_2. Dry N_2, CO_2 and SO_2 were dosed as a function of partial pressure. For the competitive binding studies with CO_2 and SO_2, the bare material was first equilibrated step by step to 1 bar of CO_2, followed by sequential replacing of CO_2 with SO_2-containing mixture (*i.e.*, tuning the SO_2/CO_2 mixture composition from 0/100 to 100/0 while maintaining a total pressure of 1 bar).

1.9 Analysis of the IAST Selectivity

Ideal adsorbed solution theory (IAST) was used to determine the selectivity factor, *S*, for binary mixtures from the pure component isotherm data. The selectivity factor, *S*, is defined according to the following Equation where x_1 is the amount of component 1 adsorbed and y_1 is the mole fraction of component 1 in the gas phase at equilibrium. The IAST adsorption selectivity was calculated for SO_2/CO_2 (1:99) and SO_2/N_2 (1:99), of compositions at 298 K and a total pressure of 1 bar.

$$\boldsymbol{S = \frac{x1/y1}{x2/y2}}$$

1.10 Powder X-ray Diffraction (PXRD) Patterns and BET Surface Analysis

Powder X-ray diffraction (PXRD) patterns were obtained using PANalytical X'Pert Pro MPD diffractometer in Bragg-Brentano geometry using Cu-Kα_1 radiation (λ = 1.5406 Å).

Volumetric cryogenic N_2, isotherms were performed on a Micromeritics 3Flex adsorption analyser using ultrahigh purity (99.999%), N_2 at 77 K for void volumetric determination. The BET surface areas were calculated using the software integrated into the instrument.

1.11 Thermo-gravimetric Analysis

Thermogravimetric analysis (SDTQ600 TA Instruments company) was used to analyse the thermostability of MFM-422. Samples were heated from room temperature to 600 °C at a heating rate of 5 °C min^{-1} under a flow of air.

2. Adsorption Isotherms

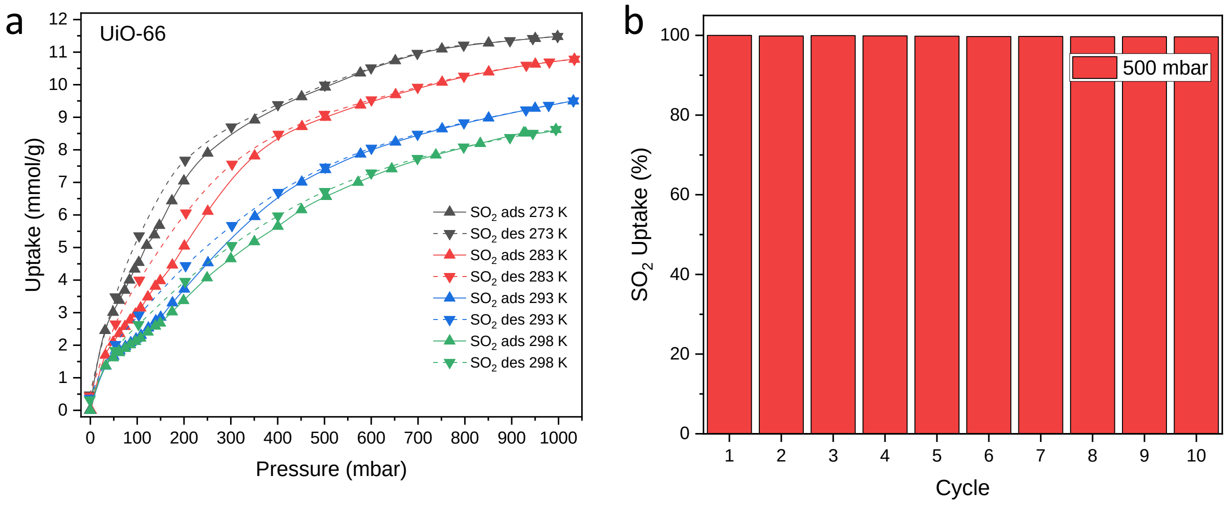

Figure S1. (a) Adsorption isotherms for SO_2 in UiO-66; (b) cycling of SO_2 between 0-500 mbar at 298 K in UiO-66.

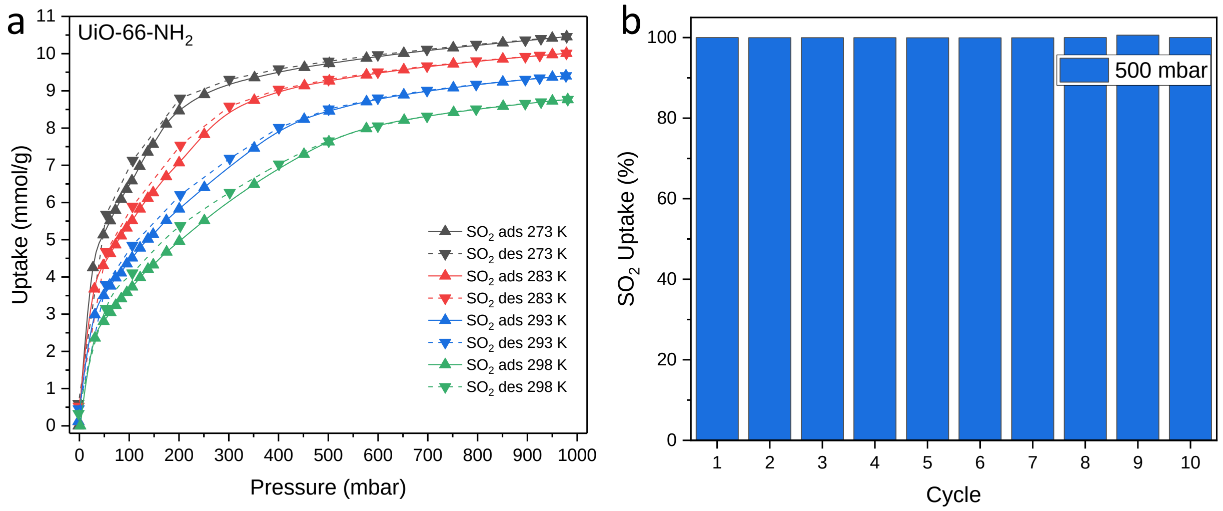

Figure S2. (a) Adsorption isotherms for SO_2 in UiO-66-NH_2; (b) cycling of SO_2 between 0-500 mbar at 298 K in UiO-66-NH_2.

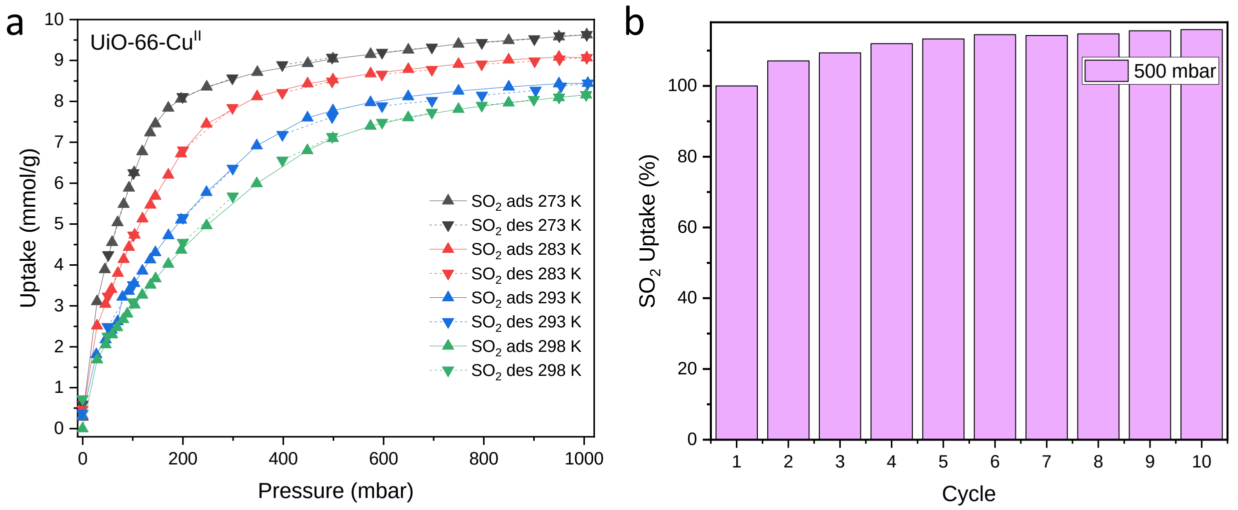

Figure S3. (a) Adsorption isotherms for SO_2 in UiO-66-Cu^{II}; (b) cycling of SO_2 between 0-500 mbar at 298 K in UiO-66-Cu^{II} (gradual increase in the uptake was due to the minor amount of retained SO_2 in UiO-66-Cu^{II} upon desorption under pressure-swing conditions).

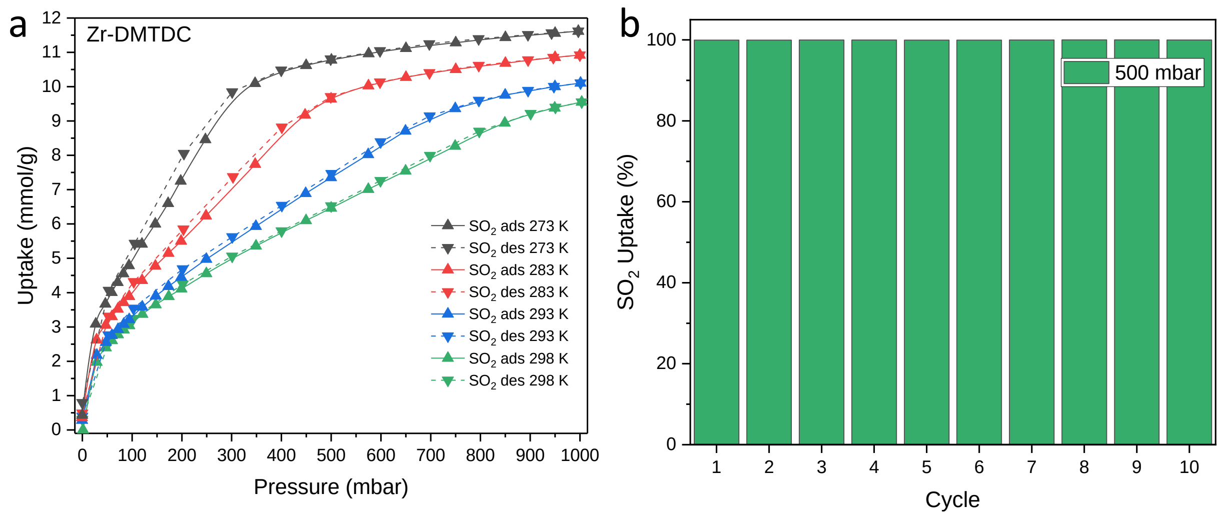

Figure S4. (a) Adsorption isotherms for SO_2 in Zr-DMTDC; (b) cycling of SO_2 between 0-500 mbar at 298 K in Zr-DMTDC.

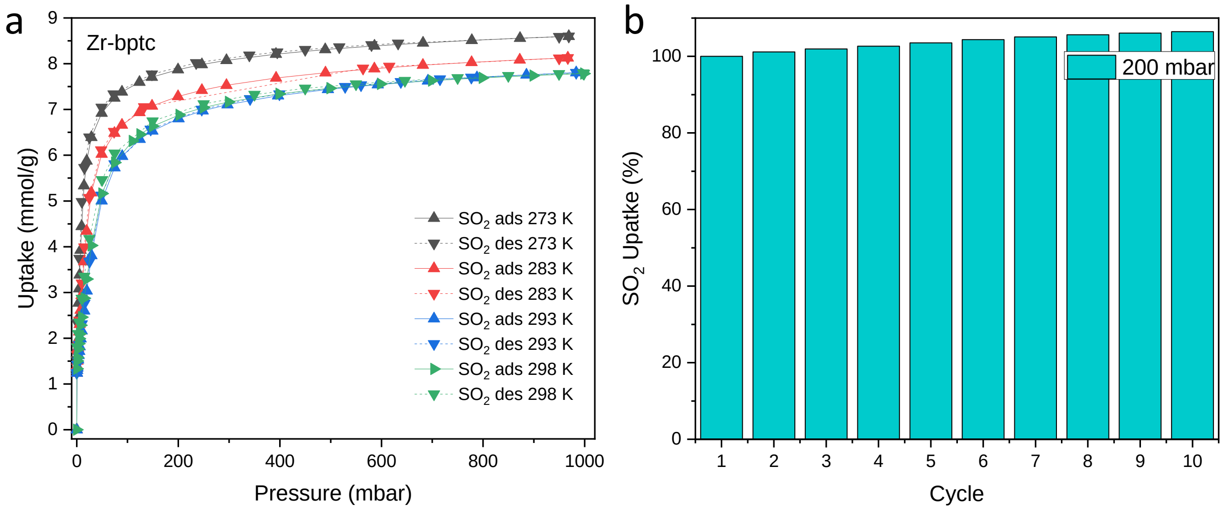

Figure S5. (a) Adsorption isotherms for SO_2 in Zr-bptc; (b) cycling of SO_2 between 0-200 mbar at 298 K in Zr-bptc (gradual increase in the uptake was due to the minor amount of retained SO_2 in UiO-66-Cu^{II} upon desorption under pressure-swing conditions).

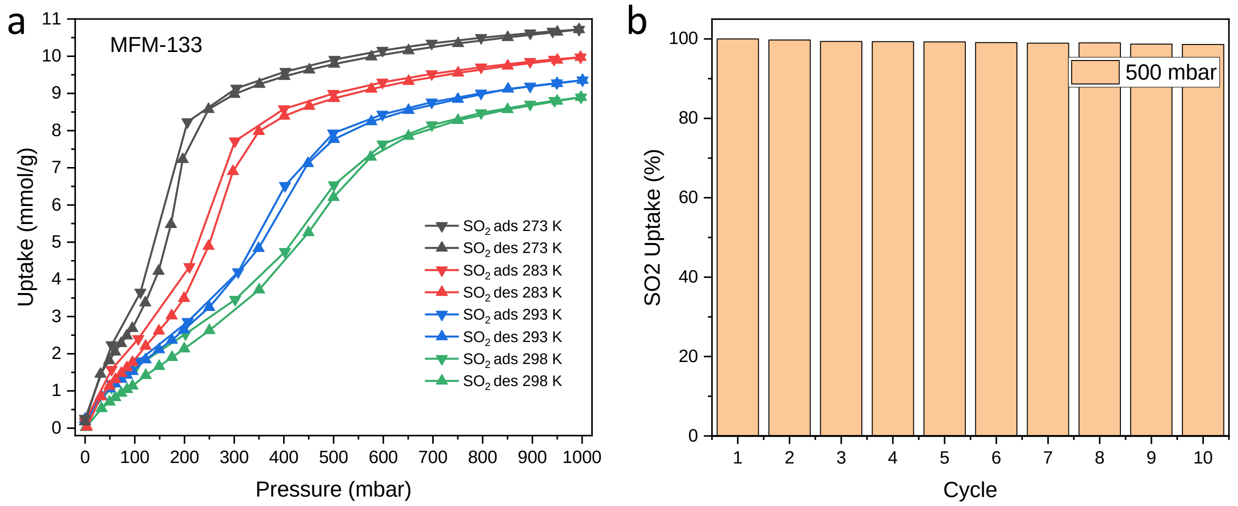

Figure S6. (a) Adsorption isotherms for SO_2 in MFM-133; (b) cycling of SO_2 between 0-500 mbar at 298 K in MFM-133.

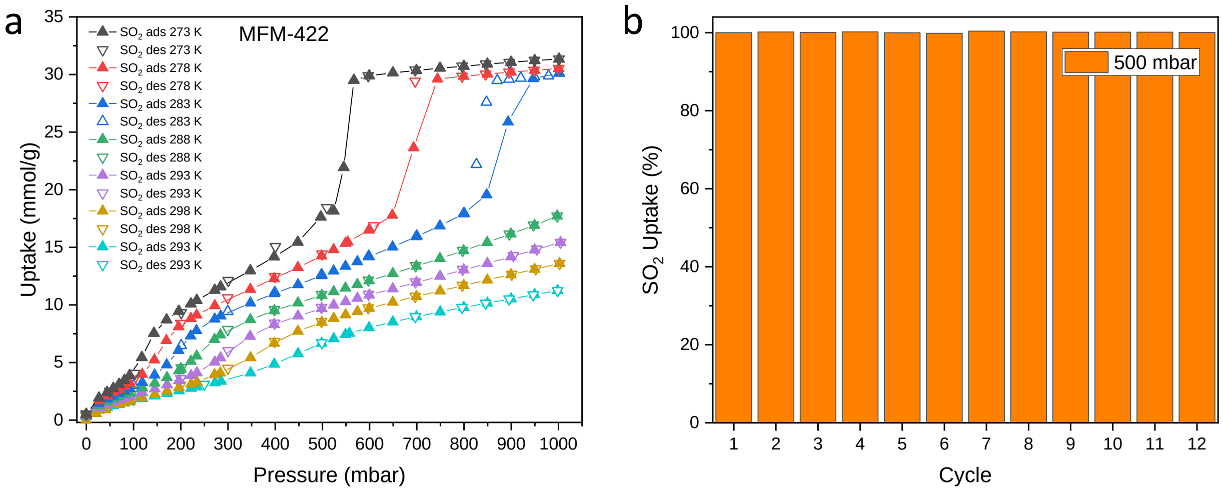

Figure S7. (a) Adsorption isotherms for SO_2 in MFM-422; (b) cycling of SO_2 between 0-500 mbar at 298 K in MFM-422.

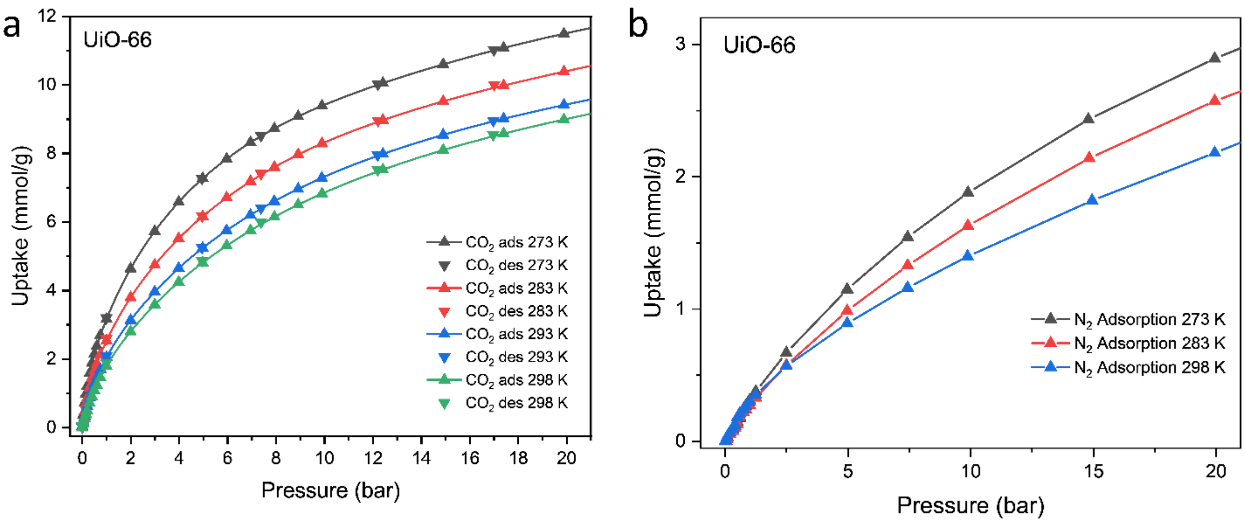

Figure S8. (a) Adsorption isotherms for CO_2 in UiO-66; (b) adsorption isotherms for N_2 in UiO-66.

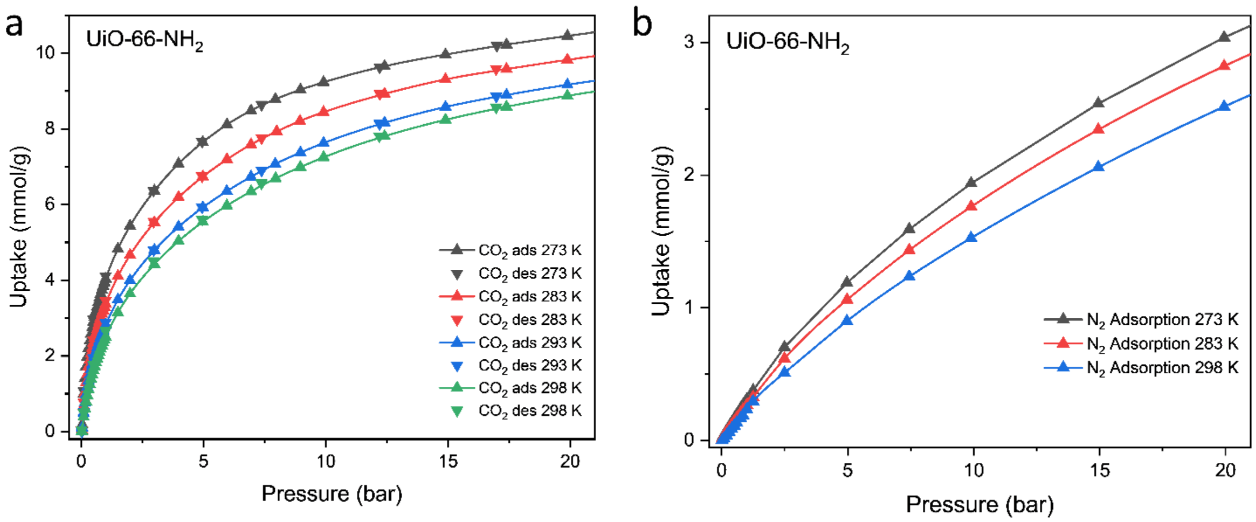

Figure S9. (a) Adsorption isotherms for CO_2 in UiO-66-NH$_2$; (b) adsorption isotherms for N_2 in UiO-66-NH$_2$.

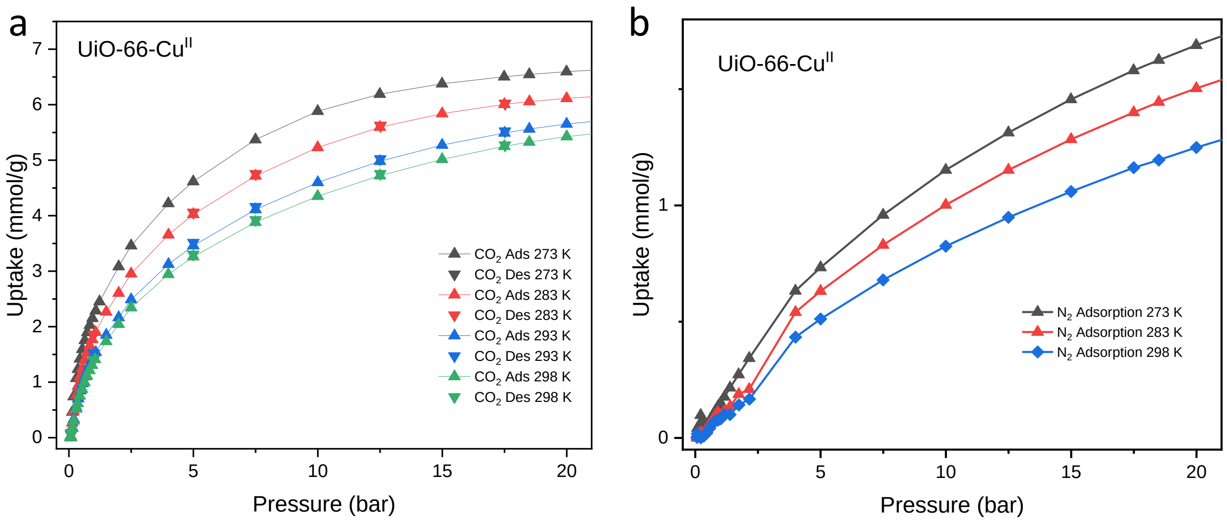

Figure S10. (a) Adsorption isotherms for CO_2 in UiO-66-Cu^{II}; (b) adsorption isotherms for N_2 in UiO-66-Cu^{II}.

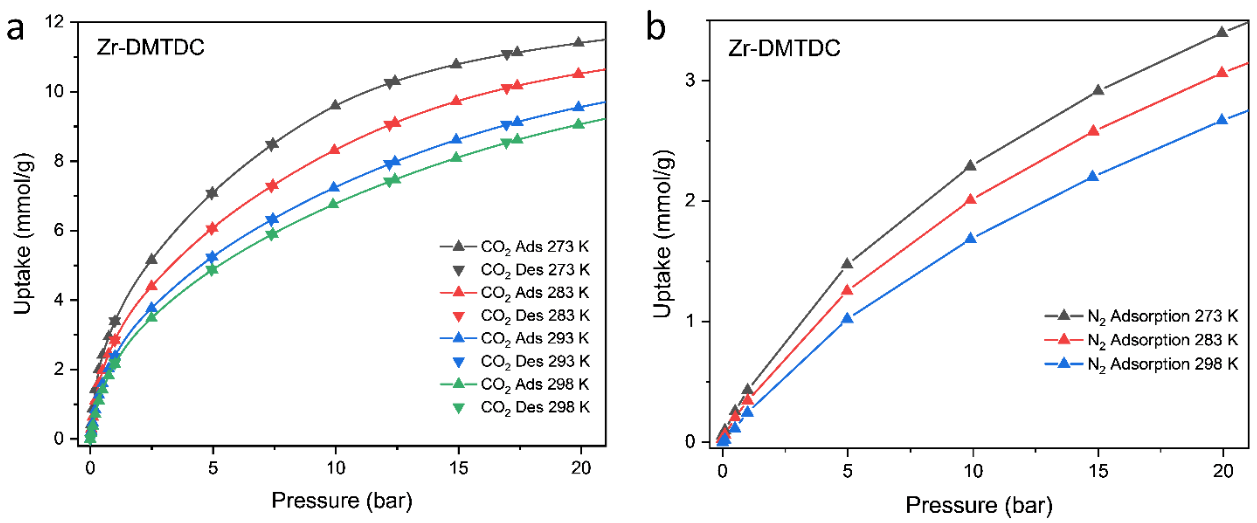

Figure S11. (a) Adsorption isotherms for CO_2 in Zr-DMTDC; (b) adsorption isotherms for N_2 in Zr-DMTDC.

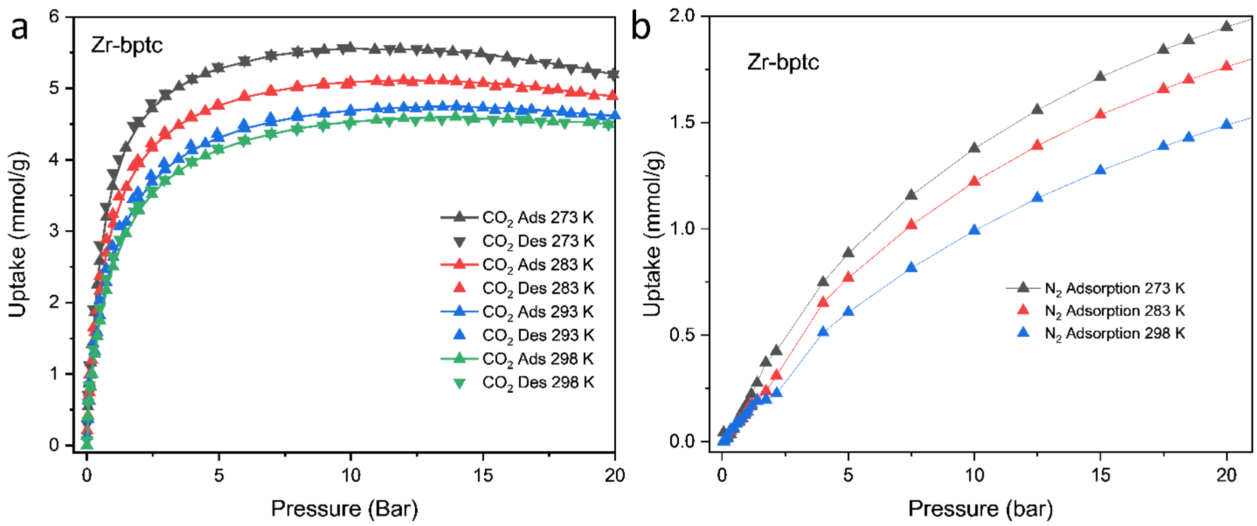

Figure S12. (a) Adsorption isotherms for CO_2 in Zr-bptc; (b) adsorption isotherms for N_2 in Zr-bptc.

Table S1. Comparison of CO_2 and N_2 uptake at 1 bar and 298 K for Zr-based MOFs

	UiO-66	UiO-66-NH_2	UiO-66-Cu^{II}	Zr-DMTDC	Zr-bptc
CO_2 (mmol g^{-1})	1.8	2.5	1.4	2.2	2.5
N_2 (mmol g^{-1})	0.3	0.2	0.08	0.2	0.1

Table S2. Summary of SO_2 adsorption in various reported solid porous materials [a]

MOF	BET Surface Area (m^2 g^{-1})	Vp (cm^3 g^{-1})	SO_2 Adsorption Capacity (mmol g^{-1})	Open Metal Site	Reference
UiO-66	1221	0.55	8.6	N	This work
UiO-66-NH_2	1037	0.52	8.8	N	This work
UiO-66-Cu^{II}	1068	0.54	8.2	N	This work
MFM-133	2156	0.96	8.9	N	This work
Zr-bptc	960	0.34	7.8	N	This work
Zr-DMTDC	1345	0.68	9.6	N	This work
MFM-422	3296	-	13.6	N	This work
MFM-170	2408	0.88	17.5	Y	15
MFM-170·H_2O	2003	n/a	13.0	N	15
SIFSIX-1-Cu	1337	n/a	11.0	N	16
[$Zn_2(L_1)_2$(bipy)]	275	0.059	10.9	N	17
MFM-202a	2220	n/a	10.2	N	18
Ni(bdc)$(ted)_{0.5}$	1783	0.74	10.0	N	19
Mg-MOF-74	1475	0.62	8.6	Y	20
MFM-300(In)	1071	0.37	8.3	N	21
SIFSIX-2-Cu-i	735	n/a	6.9	N	16
PI-COF-m	1003	n/a	6.5	n/a	22
SIFSIX-2-Cu	3140	n/a	6.5	N	16
[$Zn_2(L_1)_2$(bpe)]	379	0.081	6.4	N	17
PI-COF-m10	831	n/a	6.3	n/a	22
PI-COF-m20	548	n/a	5.6	n/a	22
PI-COF-m40	279	n/a	5.5	n/a	22
PI-COF-m60	93	n/a	4.7	n/a	22
Zn(bdc)$(ted)_{0.5}$	1888	0.84	4.4	N	19
SIFSIX-3-Ni	223	n/a	2.7	N	16
Prussian Blue	712	n/a	2.5	N	23
[Zn4(μ_4-O)$(L_1)_3$]	299	0.047	2.2	N	17
FMOF-2	378	n/a	2.2	Y	24
SIFSIX-3-Zn	250	n/a	2.1	N	16
MFM-600	2281	n/a	5.0	N	25
MFM-601	3644	n/a	12.3	N	25
MFM-305	799	0.347	7.0	N	26
MFM-305-CH_3	256	0.209	5.2	N	26
KAUST-7	280	0.095	2.6	N	27
KAUST-8	250	0.102	2.9	Y	27
MIL-101(Cr)-4F(1%)	2176	1.19	18.4	Y	28
MFM-300(Sc)	1360	0.56	9.4	N	29
MOF-177	4100	1.51	25.7*	N	30
ECUT-100	-	0.27	5.0	N	31
MFM-300(Sc)@EtOH	-	-	13.2	N	29
MOF-808-His	1054	0.523	10.4*	n	32
[Pd_6L_8]$(NO_3)_{36}$	111	n/a	6.1*	n	33
ECUT-111	1493	0.629	11.6	N	34

Cage-U-Co-MOF	208	n/a	3.6	Y	35
NOTT-401	1502	0.66	6.6	N	36
ECUT-77	760	0.40	8.0[b]	N	34
NU-1000	1972	n/a	10.9	n/a	37
[Ir]@NU-1000	1858	n/a	10.6	n/a	37
Fe-soc-MOF	1470	0.58	11.7*	Y	38
CAU-10	630	0.25	4.5	N	39
CPL-1	335	0.125	2.0	N	40
ELM-12	706	0.26	2.7	n/a	41
MOF-808	2380	1.1	15.3	N	42
EDTA-MOF-808	1036	0.47	9.8	N	42
[RuGa]@NU-1000	1796	n/a	7.5	N	43
CB_6@MIL-101-Cl	2077	1.0	17.0	n/a	44
MIP-206-OH	-	0.45	5.9	N	45
Zr-Fum	600	0.290	4.9[c]	n/a	46
DUT-67(Zr)	1260	0.544	9.0[c]	n/a	46
MIL-53(Al)	1450	0.706	10.5[c]	n/a	46
Al-Fum	970	0.447	7.5[c]	n/a	46
MIL-53(tdc)(Al)	1000	0.415	6.9[c]	n/a	46
CAU-10-H	600	0.258	4.8[c]	n/a	46
MIL-96(Al)	530	0.237	6.5[c]	n/a	46
MIL-100(Al)	1890	0.824	16.3[c]	n/a	46
DMOF	1956	0.75	13.1 [c,d]	n/a	47
DMOF-M	1557	0.63	12.2 [c,d]	n/a	47
DMOF-DM	1343	0.52	10.4 [c,d]	n/a	47
DMOF-TM	900	0.43	9.7 [c,d]	n/a	47

[a.] The uptake was recorded at 298 K and 1 bar; [b.] the uptake was recorded to 0.92 bar; [c.] the uptake was recorded at 293 K; [d.] the uptake was recorded to 0.97 bar; *the MOF is unstable towards SO_2 desorption.

3. Selectivity Data for Zr-MOFs

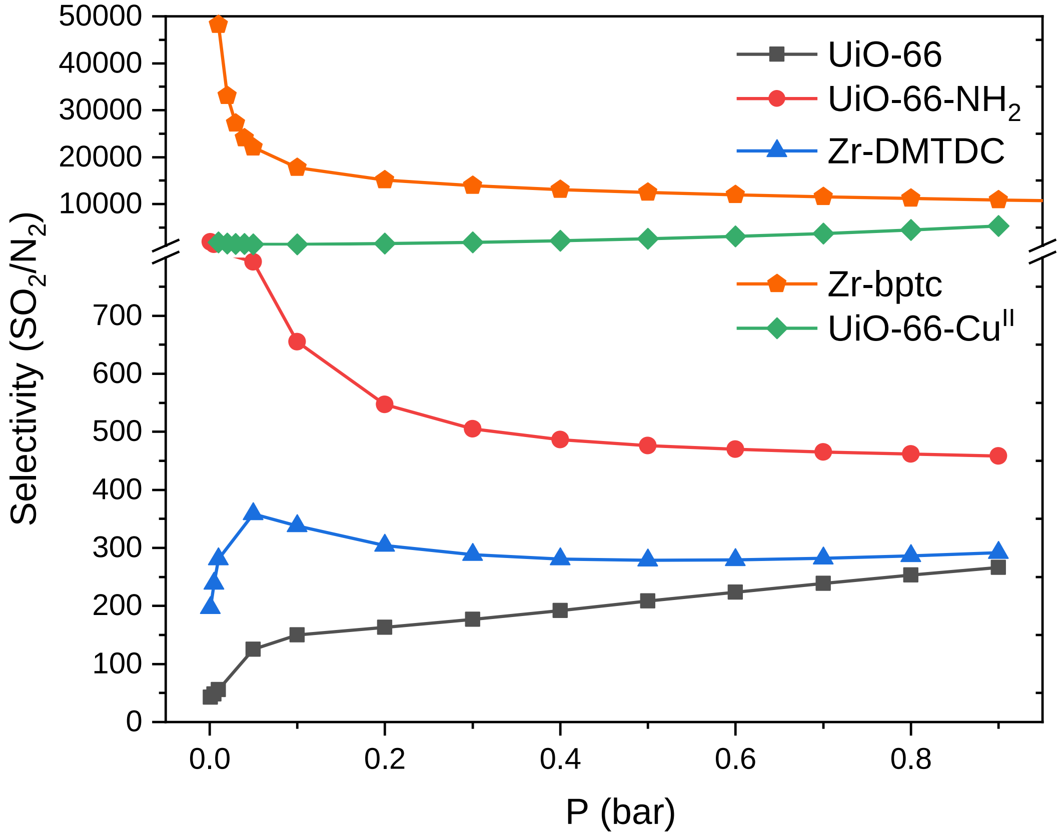

Figure S13. IAST selectivity for mixture of SO_2/N_2 (1:99) for UiO-66, UiO-66-NH_2, UiO-66-Cu^{II}, Zr-DMTDC and Zr-bptc at 298 K.

4. *In situ* Synchrotron X-Ray Powder Diffraction

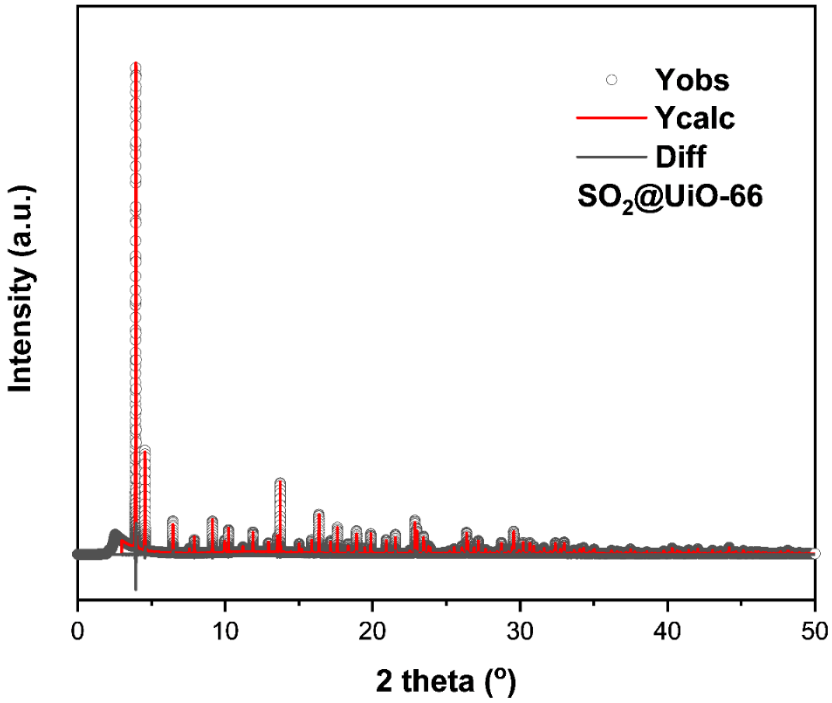

Figure S14. PXRD patterns [observed (black), calculated (red) and difference (grey)] for the Rietveld refinement of $Zr_6O_4(OH)_4(bdc)_6{\cdot}(SO_2)_{7.7}$.

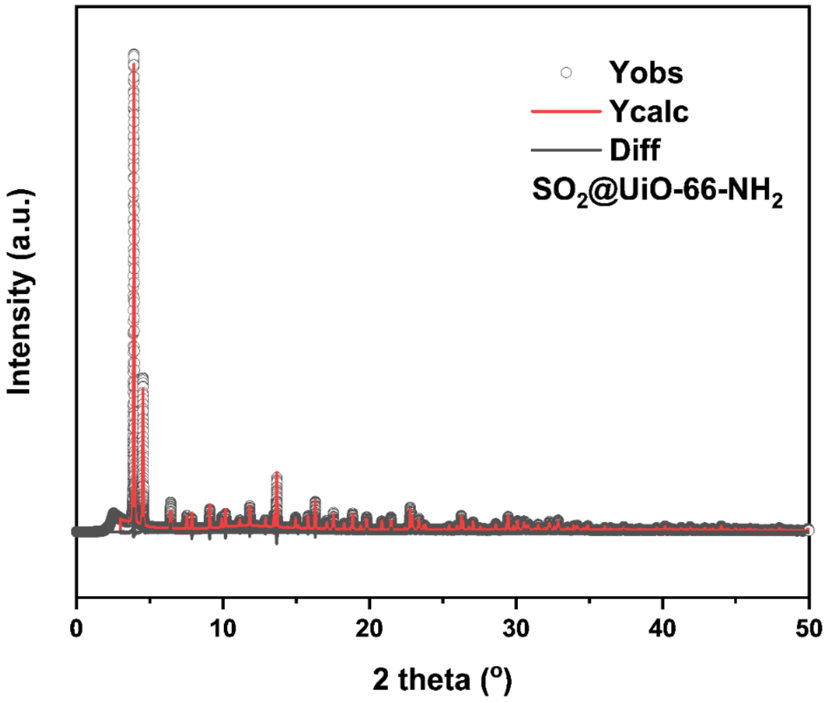

Figure S15. PXRD patterns [observed (black), calculated (red) and difference (grey)] for the Rietveld refinement of [$Zr_6O_4(OH)_4(bdc\text{-}NH_2)_6{\cdot}(SO_2)_{8.1}$].

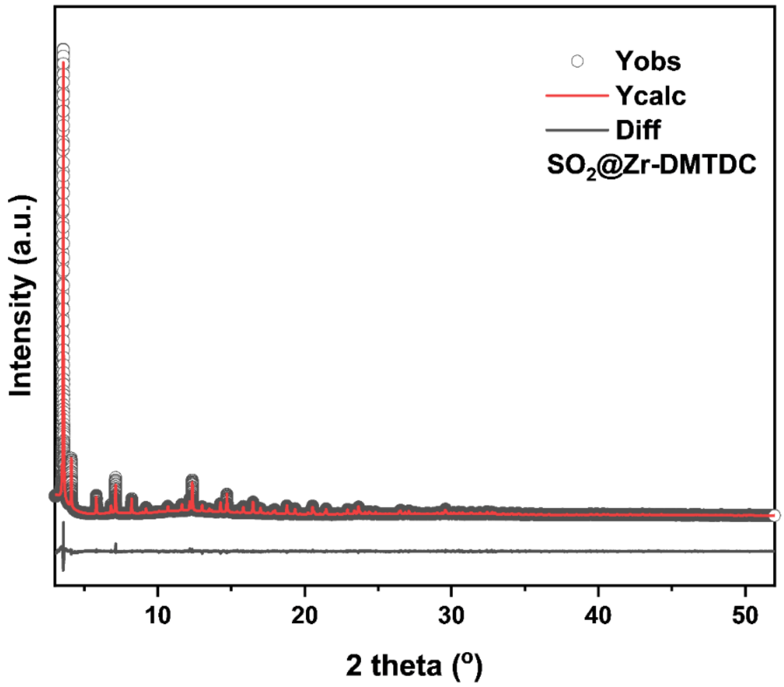

Figure S16. PXRD patterns [observed (black), calculated (red) and difference (grey)] for the Rietveld refinement of $[Zr_6O_4(OH)_4(DMTDC)_6{\cdot}(SO_2)_{13.1}]$.

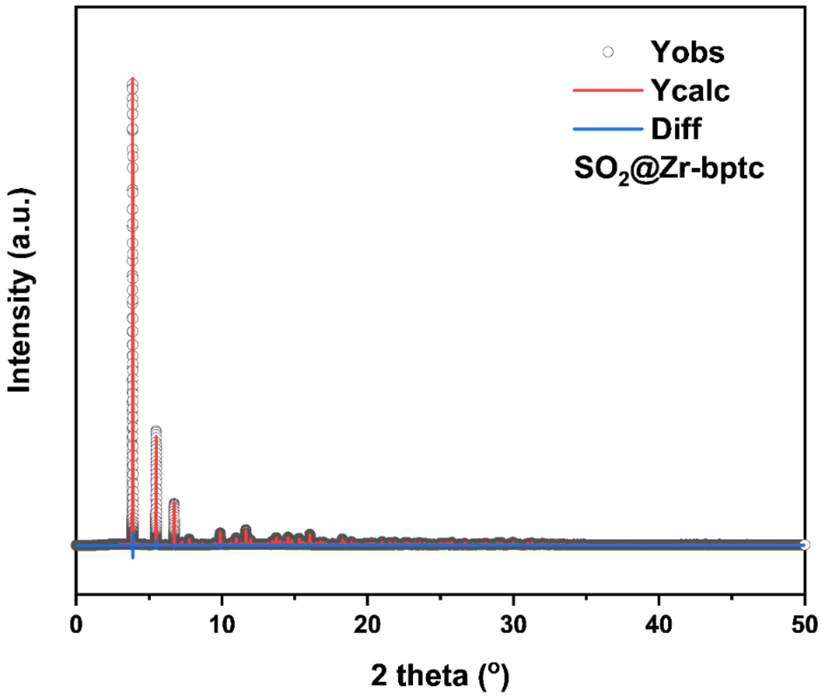

Figure S17. PXRD patterns [observed (black), calculated (red) and difference (grey)] for the Rietveld refinement of $[Zr_6O_4(OH)_4(bptc)_3{\cdot}(SO_2)_{5.8}]$.

Table S3. Summary of powder X-ray diffraction refinements for gas loaded MOFs

	**SO_2@UiO-66**	**SO_2@UiO-66-NH_2**	**SO_2@Zr-DMTDC**	**SO_2@Zr-bptc**
**Formula**	$[Zr_6O_4(OH)_4(bdc)_2\cdot(SO_2)_{7.7}]$,	$[Zr_6O_4(OH)_4(bdc$-$NH_2)_2\cdot(SO_2)_{8.1}]$,	$[Zr_6O_4(OH)_4(DMTDC)_2\cdot(SO_2)_{13.1}]$,	$[Zr_6O_4(OH)_4(bptc)_3\cdot(SO_2)_{1.2}]$
**Formula weight (g/mol)**	2150.897	2217.630	3082.826	2025.512
**Temp (K)**	298			
**Radiation type**	Synchrotron			
**Diffractometer**	Beamline I11 of Diamond Light Source			
**Data collection mode**	Transmission			
**Wavelength (Å)**	0.8268(3)	0.8268(3)	0.8260(1)	0.8245(2)
**Crystal system**	cubic	cubic	cubic	orthorhombic
**Space group**	Fm-3m	Fm-3m	F-43m	Immm
**a / Å**	20.76(1)	20.76(1)	23.06(2)	24.40(7)
**b / Å**	20.76(1)	20.76(1)	23.06(2)	24.29(4)
**c / Å**	20.76(1)	20.76(1)	23.06(2)	24.39(6)
**α/ β/ γ**	90	90	90	90
**V / Å^3**	8,947.5(3)	8,947.5(3)	12,266.3(2)	14,465.0(4)
**D_c g/cm^3**	1.598	1.599	1.669	1.873
**R_{exp} / %**	3.171	3.872	3.171	2.934
**R_{wp} / %**	6.944	8.428	6.945	7.312
**R_p / %**	5.091	6.064	5.091	5.029
GoF	2.190	2.177	2.190	2.492
**R_{Bragg}**	3.281	5.394	3.281	2.472

Table S4. Summary of crystallographic data of MFM-422

	**MFM-422**
Formula	$C_{46}H_{26}O_{16}Zr_3$
Formula Weight	1108.36
Crystal System	hexagonal
Space group	*P6/mmm*
a, Å<br>*c*, Å	40.1818(16)<br>20.1586(7)
V, Å^3	28187(2)
Z	6
D_c, g cm^{-3}	0.392
μ, mm^{-1}	0.167
Crystal size, mm	0.01 x 0.02 x 0.01
Temperature, K	120
Radiation wavelength, Å	0.68890
θ range, °	1.6 – 16.9
Reflections collected/ unique	6333, 3070
Reflections with $I > 2\sigma(\mathrm{I})$	2350
R_{int}	0.272
$R[F^2 > 2\sigma(F^2)]$, $wR(F^2)$, S	0.0779, 0.2261, 0.892
Number of reflections	6333
Number of parameters	170
$\Delta\rho_{max}$/ $\Delta\rho_{min}$,eÅ^{-3}	-0.829, 0.705
CCDC Deposit Number	2132832

5. *In situ* Infrared Spectroscopy

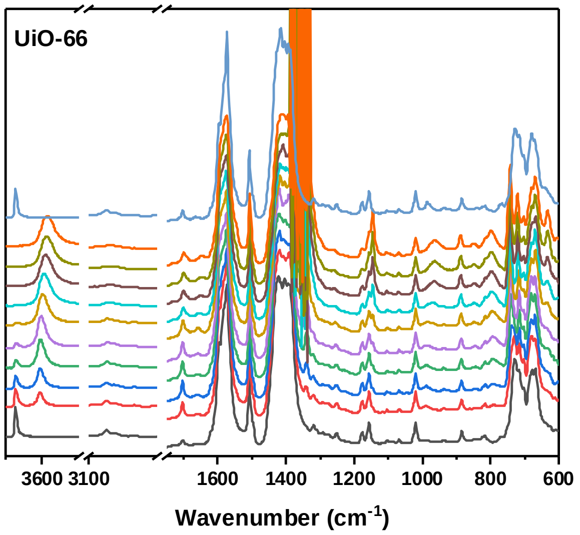

Figure S18. *In situ* FTIR spectra of SO_2@UiO-66 at 298 K. From bottom to top: activated UiO-66, 1% SO_2-loading, 2% SO_2-loading, 5% SO_2-loading, 10% SO_2-loading, 20% SO_2-loading, 40% SO_2-loading, 60% SO_2-loading, 80% SO_2-loading, 100% SO_2-loading and regenerated UiO-66.

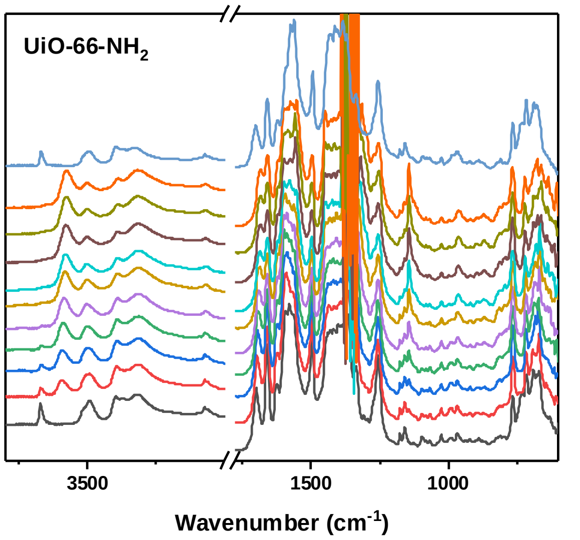

Figure S19. *In situ* FTIR spectra of SO_2@UiO-66-NH_2 at 298 K. From bottom to top: activated UiO-66-NH_2, 1% SO_2-loading, 2% SO_2-loading, 5% SO_2-loading, 10% SO_2-loading, 20% SO_2-loading, 40% SO_2-loading, 60% SO_2-loading, 80% SO_2-loading, 100% SO_2-loading and regenerated UiO-66-NH_2.

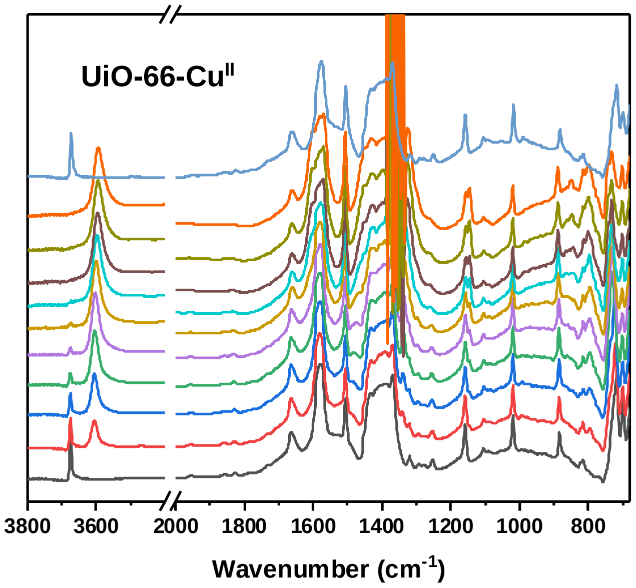

Figure S20. *In situ* FTIR spectra of SO_2@UiO-66-Cu^{II} at 298 K. From bottom to top: activated UiO-66, 1% SO_2-loading, 2% SO_2-loading, 5% SO_2-loading, 10% SO_2-loading, 20% SO_2-loading, 40% SO_2-loading, 60% SO_2-loading, 80% SO_2-loading, 100% SO_2-loading and regenerated UiO-66-Cu^{II}.

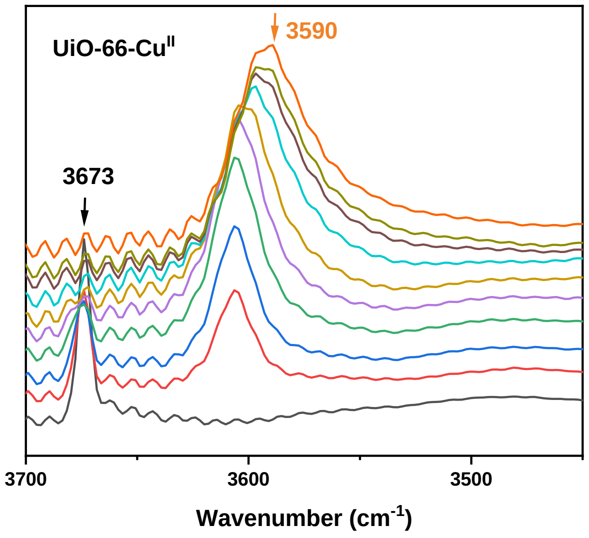

Figure S21. IR spectra of the $\nu(\mu_3\text{-OH})$ peak in SO_2@UiO-66-Cu^{II} at 298 K. From bottom to top: activated UiO-66-Cu^{II}, 1% SO_2-loading, 2% SO_2-loading, 5% SO_2-loading, 10% SO_2-loading, 20% SO_2-loading, 40% SO_2-loading, 60% SO_2-loading, 80% SO_2-loading, 100% SO_2-loading.

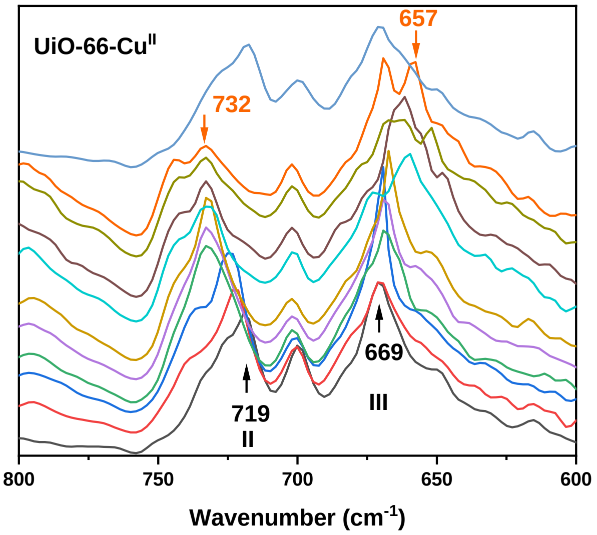

Figure S22. IR spectra of the *v*(Cu-OH) and *v*(Cu-O) stretch in SO_2@UiO-66-Cu^{II} at 298 K. From bottom to top: activated UiO-66-Cu^{II} (black), 1% SO_2-loading, 2% SO_2-loading, 5% SO_2-loading, 10% SO_2-loading, 20% SO_2-loading, 40% SO_2-loading, 60% SO_2-loading, 80% SO_2-loading, 100% SO_2-loading and regenerated UiO-66-Cu^{II}.

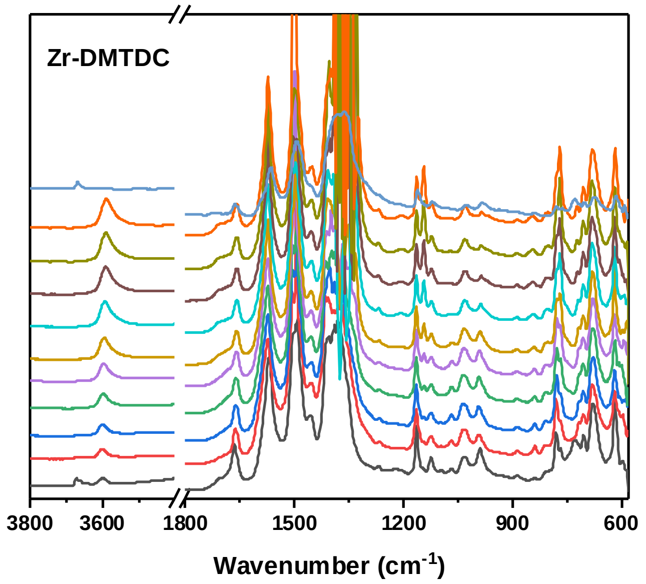

Figure S23. *In situ* FTIR spectra of SO_2@Zr-DMTDC at 298 K. From bottom to top: activated Zr-DMTDC, 1% SO_2-loading, 2% SO_2-loading, 5% SO_2-loading, 10% SO_2-loading, 20% SO_2-loading, 40% SO_2-loading, 60% SO_2-loading, 80% SO_2-loading, 100% SO_2-loading and regenerated Zr-DMTDC.

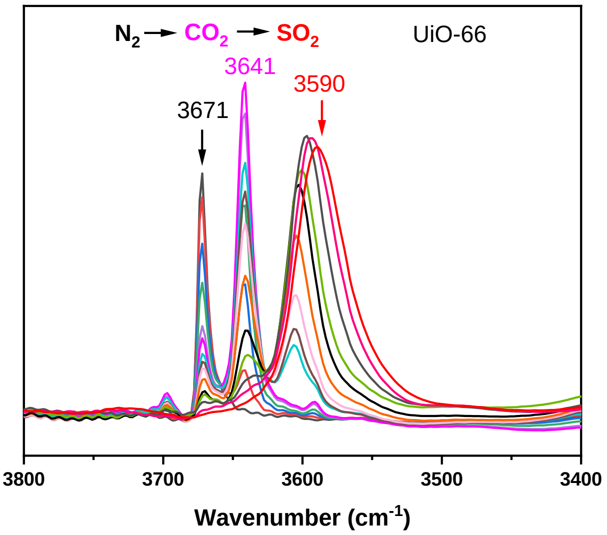

Figure S24. IR spectra of the *v*(μ_3-OH) peak for CO_2- and SO_2-loading and CO_2 displacement by SO_2 in UiO-66. Activated UiO-66 (dark grey), 5% CO_2-loading (crimson), 20% CO_2-loading (blue), 40% CO_2-loading (sea green), 80% CO_2-loading (violet), 100% CO_2-loading (magenta); 1% SO_2-loading (cyan), 2% SO_2-loading (light wine), 3% SO_2-loading (rose), 5% SO_2-loading (orange), 10% SO_2-loading (black), 15% SO_2-loading (light olive), 30% SO_2-loading (light black), 50% SO_2-loading (pink) and 80% SO_2-loading (red).

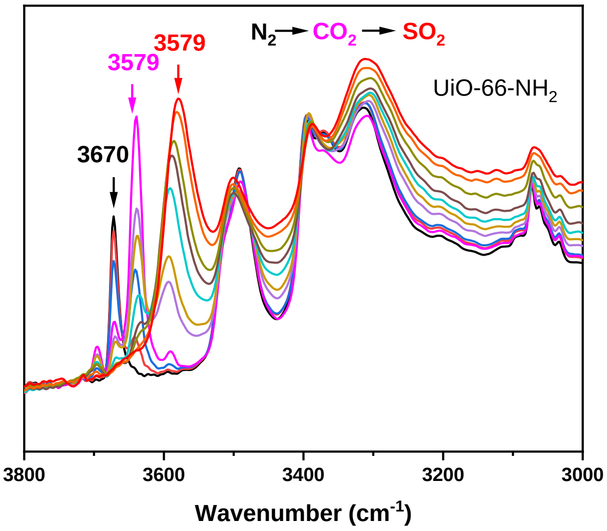

Figure S25. IR spectra of the *v*(μ_3-OH) peak for CO_2- and SO_2-loading and CO_2 displacement by SO_2 in UiO-66-NH_2. Activated UiO-66-NH_2 (black), 5% CO_2-loading (crimson), 20% CO_2-loading (dark blue), 100% CO_2-loading (magenta); 1% SO_2-loading (violet), 2% SO_2-loading (light orange), 5% SO_2-loading (cyan), 10% SO_2-loading (wine), 20% SO_2-loading (dark olive green), 40% SO_2-loading (orange) and 60% SO_2-loading (red).

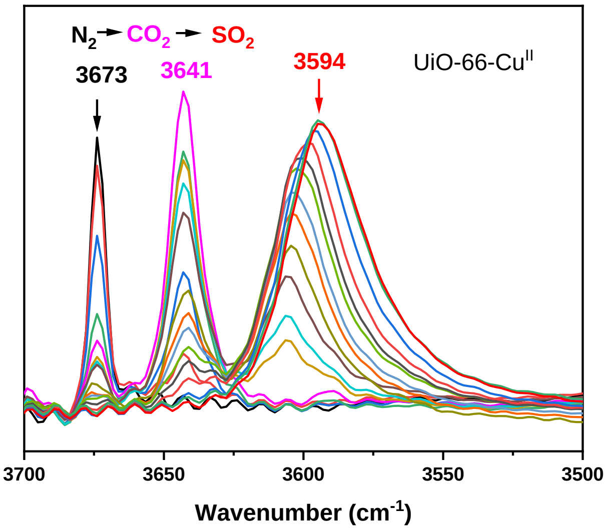

Figure S26. IR spectra of the *ν*(μ_3-OH) peak for CO_2- and SO_2-loading and CO_2 displacement by SO_2 in UiO-66-Cu^{II}. Activated UiO-66-Cu^{II} (black), 5% CO_2-loading (dark orange), 20% CO_2-loading (light blue), 100% CO_2-loading (magenta); 1% SO_2-loading (dark goldenrod), 2% SO_2-loading (aqua), 3% SO_2-loading (wine), 5% SO_2-loading (dark olive green), 7% SO_2-loading (orange), 10% SO_2-loading (cyan), 15% SO_2-loading (light green), 20% SO_2-loading (dark grey), 30% SO_2-loading (crimson), 50% SO_2-loading (blue), 80% SO_2-loading (green) and 90% SO_2-loading (red).

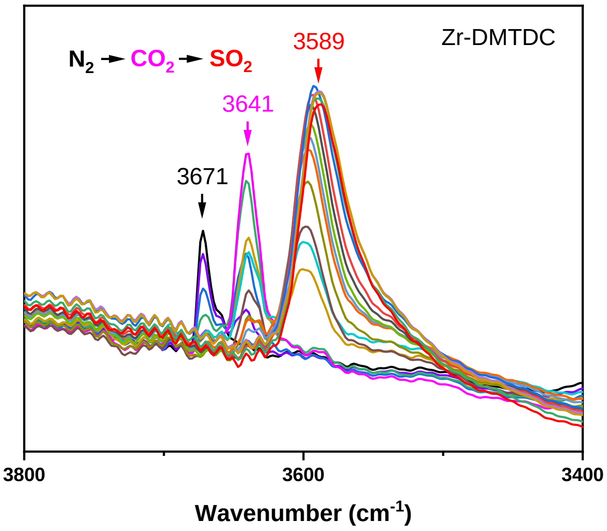

Figure S27. IR spectra of the *ν*(μ_3-OH) peak for CO_2- and SO_2-loading and CO_2 displacement by SO_2 in Zr-DMTDC. Activated UiO-66 (black), 5% CO_2-loading (dark yellow), 20% CO_2-loading (light black), 60% CO_2-loading (sea green), 100% CO_2-loading (magenta); 1% SO_2-loading (light orange), 2% SO_2-loading (dark cyan), 3% SO_2-loading (light wine), 5% SO_2-loading (dark yellow), 7% SO_2-loading (orange), 10% SO_2-loading (sea cyan), 15% SO_2-loading (dark green), 20% SO_2-loading (dark grey), 30% SO_2-loading (light red), 45% SO_2-loading (blue), 80% SO_2-loading (light violet), 90% SO_2-loading (violet), 95% SO_2-loading (dark goldenrod) and 100% SO_2-loading (red).

6. *In situ* Inelastic Neutron Scattering

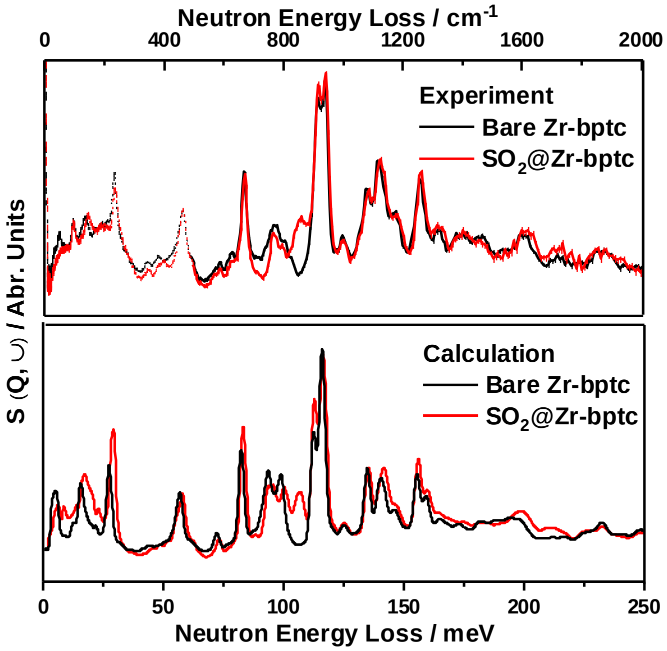

Figure S28. Experimental and DFT-calculated INS spectra of bare and SO_2-loaded Zr-bptc

7. PXRD patterns for Zr-MOFs

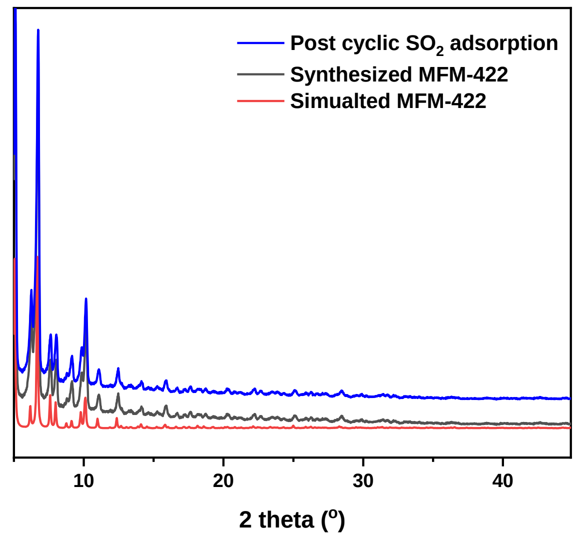

Figure S29. PXRD patterns for MFM-422.

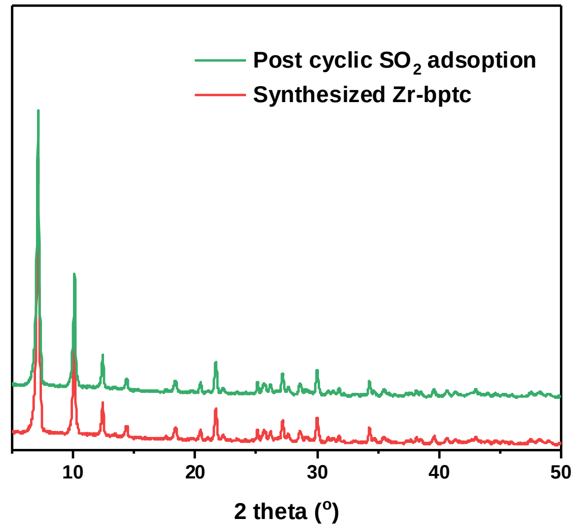

Figure S30. PXRD patterns for Zr-bptc.

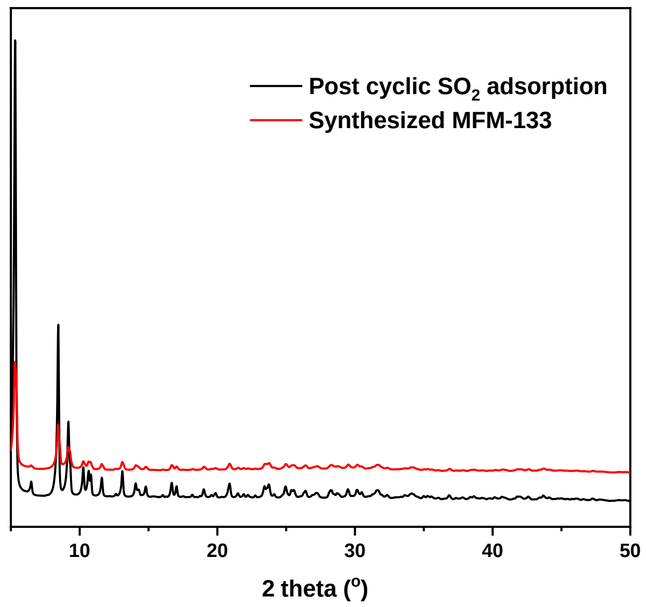

Figure S31. PXRD patterns for MFM-133.

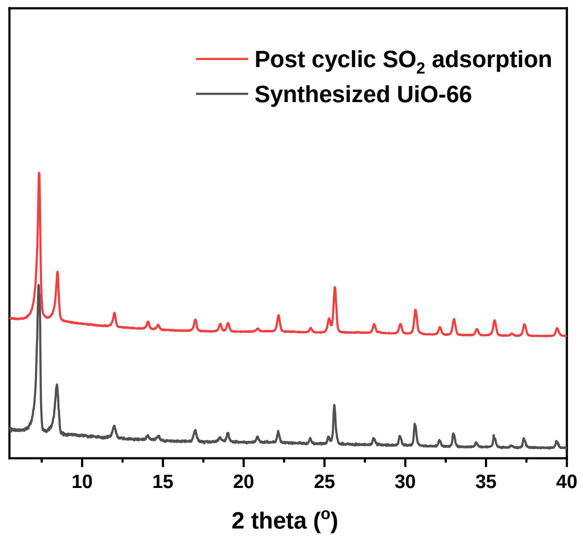

Figure S32. PXRD patterns for UiO-66.

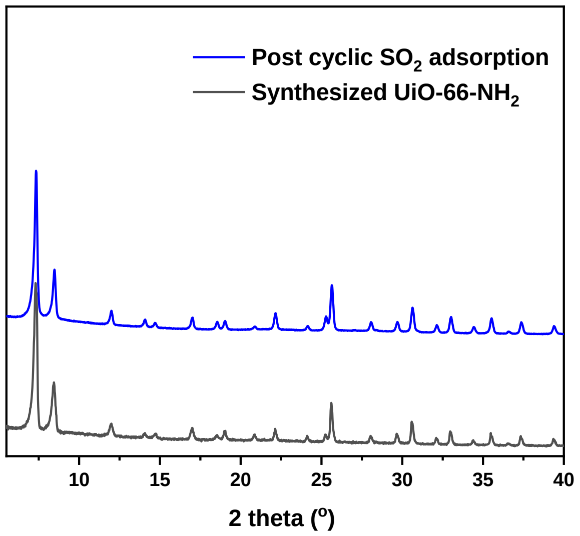

Figure S33. PXRD patterns for UiO-66-NH_2.

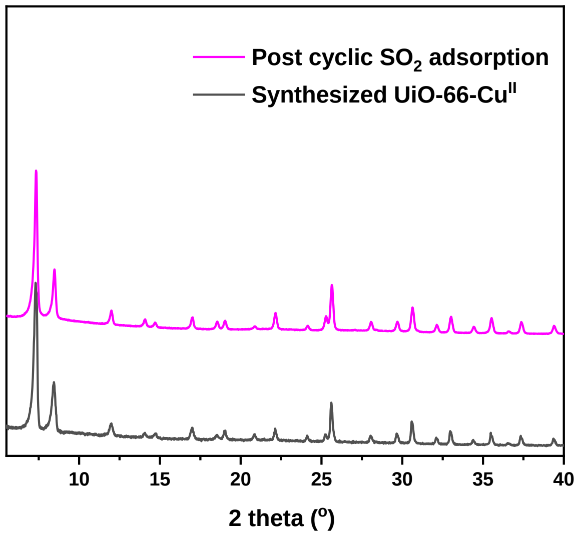

Figure S34. PXRD patterns for UiO-66-Cu^{II}.

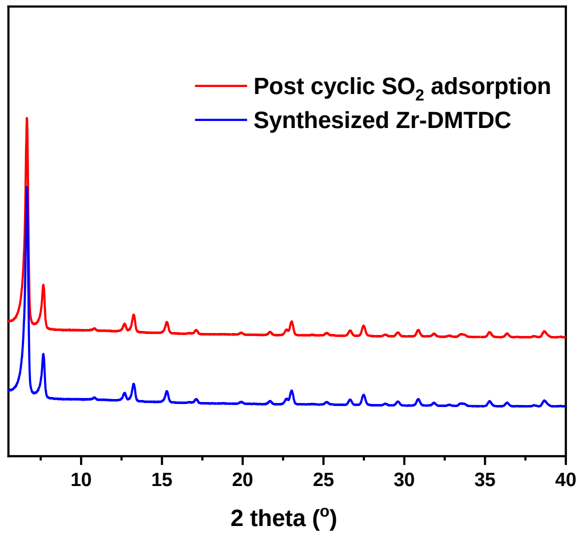

Figure S35. PXRD patterns for Zr-DMTDC.

8. Calculation of Isosteric Heats of Adsorption

To estimate the differential enthalpies (ΔH_n) and (ΔS_n) for SO_2 adsorption, all isotherms at different temperatures were fitted to the van't Hoff isochore:

$$\ln(P) = \frac{\Delta Hn}{RT} - \frac{\Delta Sn}{R}$$

where *P* is pressure, *T* is the temperature, *R* is the real gas constant. Selected linear fitting plots are shown in Figure S.36 and 42. All linear fittings show R^2 above 0.9, indicating consistency for the isotherm data. A plot of ln(P) versus 1/T at constant amount adsorbed allows the differential enthalpy of adsorption and also the isosteric enthalpy of adsorption (Q_{st}) to be determined.

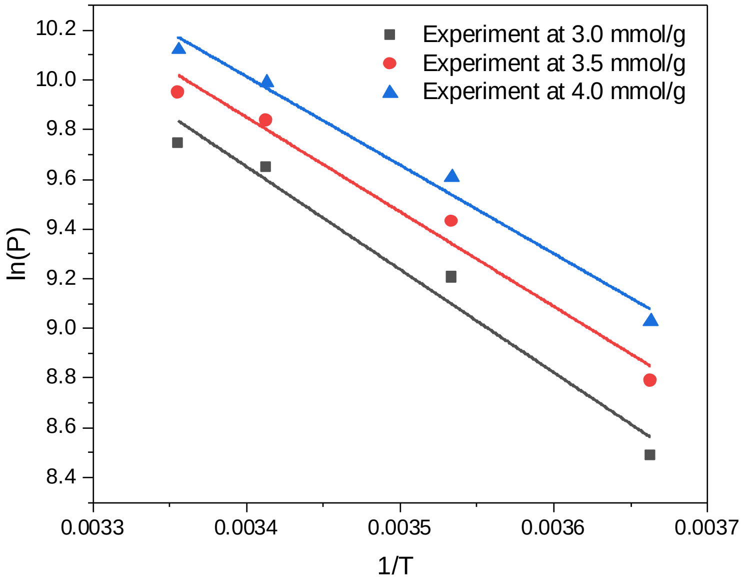

Figure S36. Linear fitting of van't Hoff plots for the SO_2 adsorption isotherms of UiO-66.

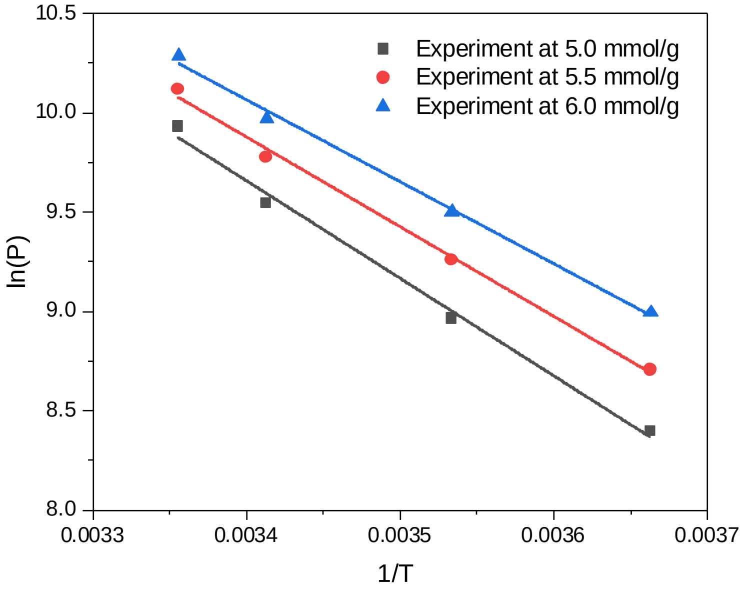

Figure S37. Linear fitting of van't Hoff plots for the SO_2 adsorption isotherms of UiO-66-NH_2.

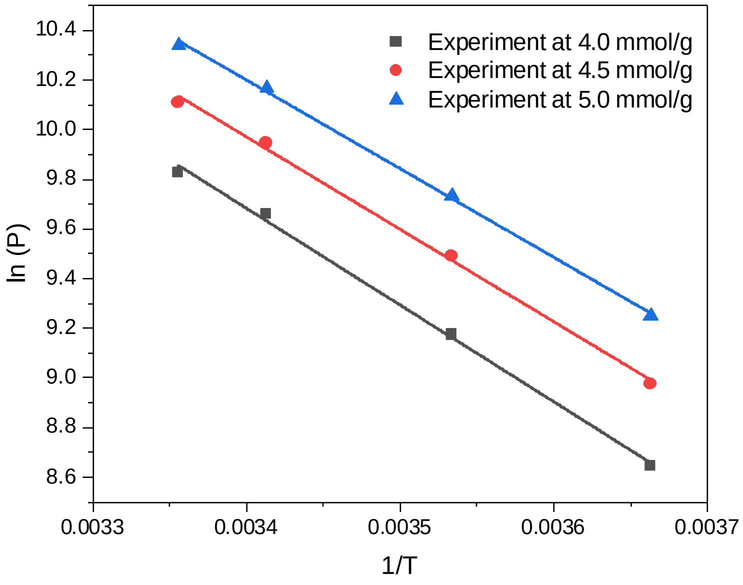

Figure S38. Linear fitting of van't Hoff plots for the SO_2 adsorption isotherms of Zr-DMTDC.

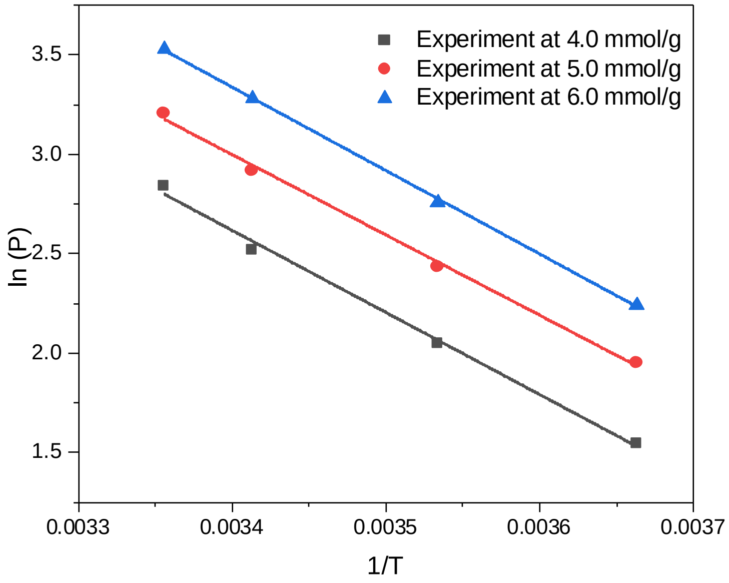

Figure S39. Linear fitting of van't Hoff plots for the SO_2 adsorption isotherms of UiO-66-Cu^{II}.

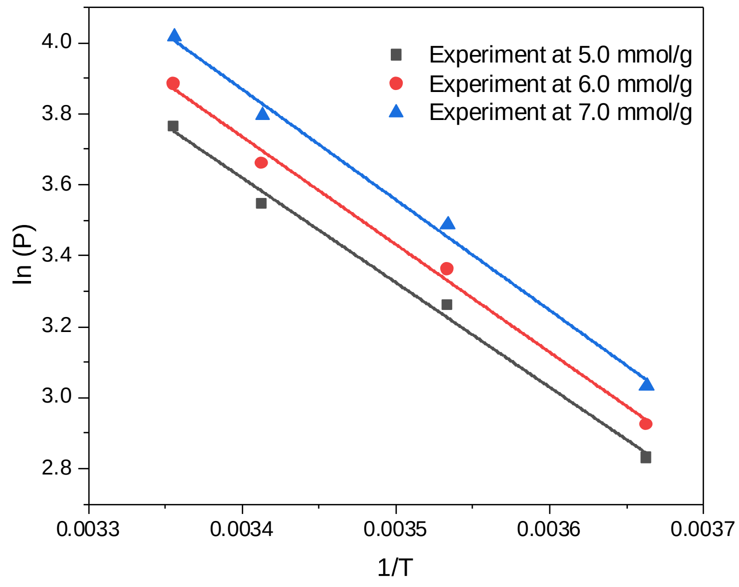

Figure S40. Linear fitting of van't Hoff plots for the SO_2 adsorption isotherms of MFM-133.

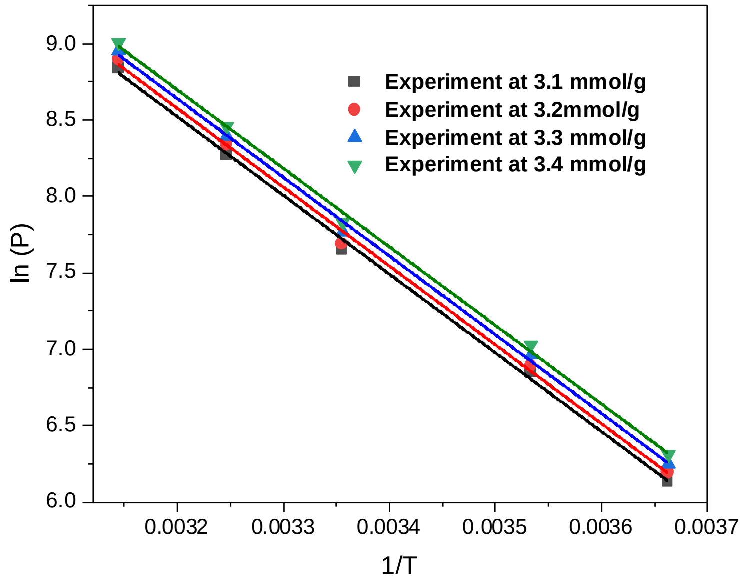

Figure S41. Linear fitting of van't Hoff plots for the SO_2 adsorption isotherms of Zr-bptc.

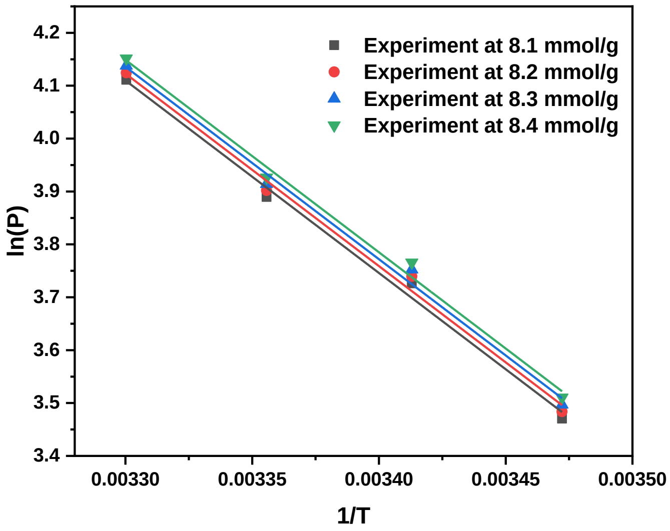

Figure S42. Linear fitting of van't Hoff plots for the SO_2 adsorption isotherms of MFM-422.

9. N_2 isotherms at 77 K of MFM-422

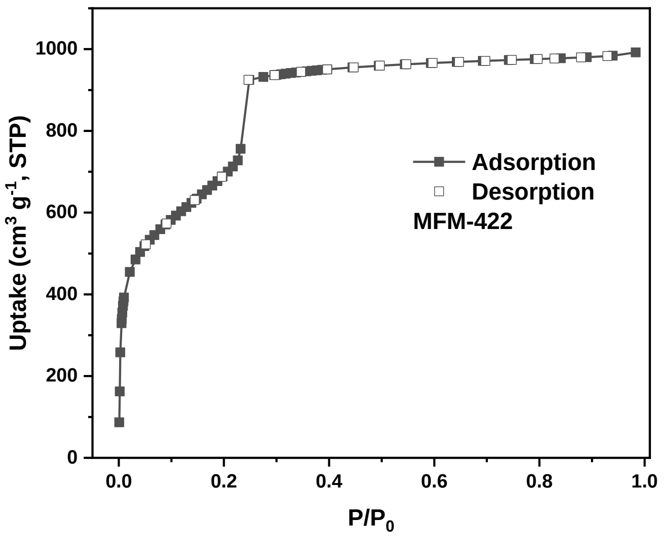

Figure S43. N_2 adsorption isotherm in MFM-422 at 77 K.

10. Thermo-gravimetric Analysis

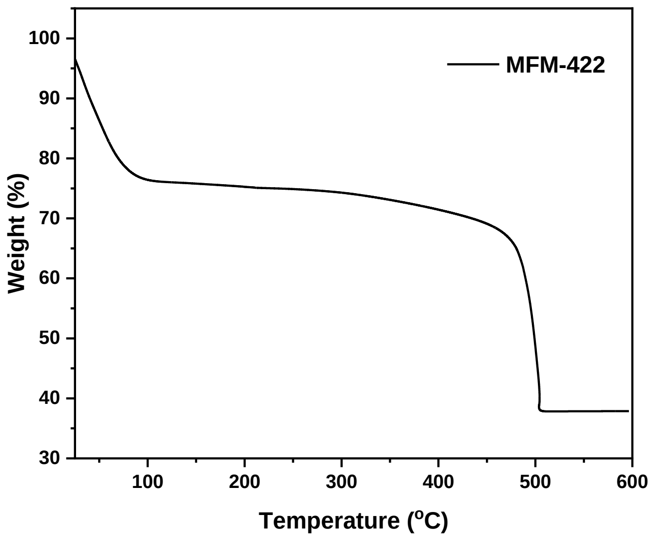

Figure S44. TGA plots of as-synthesized MFM-422. The measurements were carried out under a flow of air at a rate of 100 mL min^{-1}.

11. Conversion of Captured SO_2

Activated Zr-bptc (168 mg) was dosed with SO_2 for 1 h at 298 K to reach adsorption equilibrium (equivalent to 1.31 mmol SO_2), then morpholin-4-amine (128.0 mg, 1.25 mmol) and CH_3CN (3 mL) were added and stirred for 1 h. 4-Methoxy-aryldiazonium tetrafluoroborate (55.0 mg, 0.25 mmol) in CH_3CN (1 mL) was added dropwise to the above suspension, and the mixture was stirred at room temperature for 1 h. The mixture was centrifuged, and the supernatant was evaporated. NMR spectroscopy and preparative thin layer chromatography (TLC) were used to quantify the conversion of 4-methoxy-aryldiazonium tetrafluoroborate and the yield of the sulfonamide.

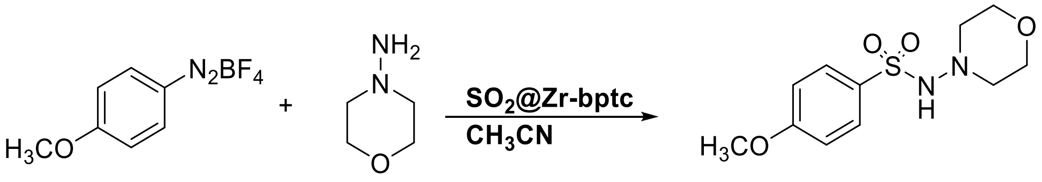

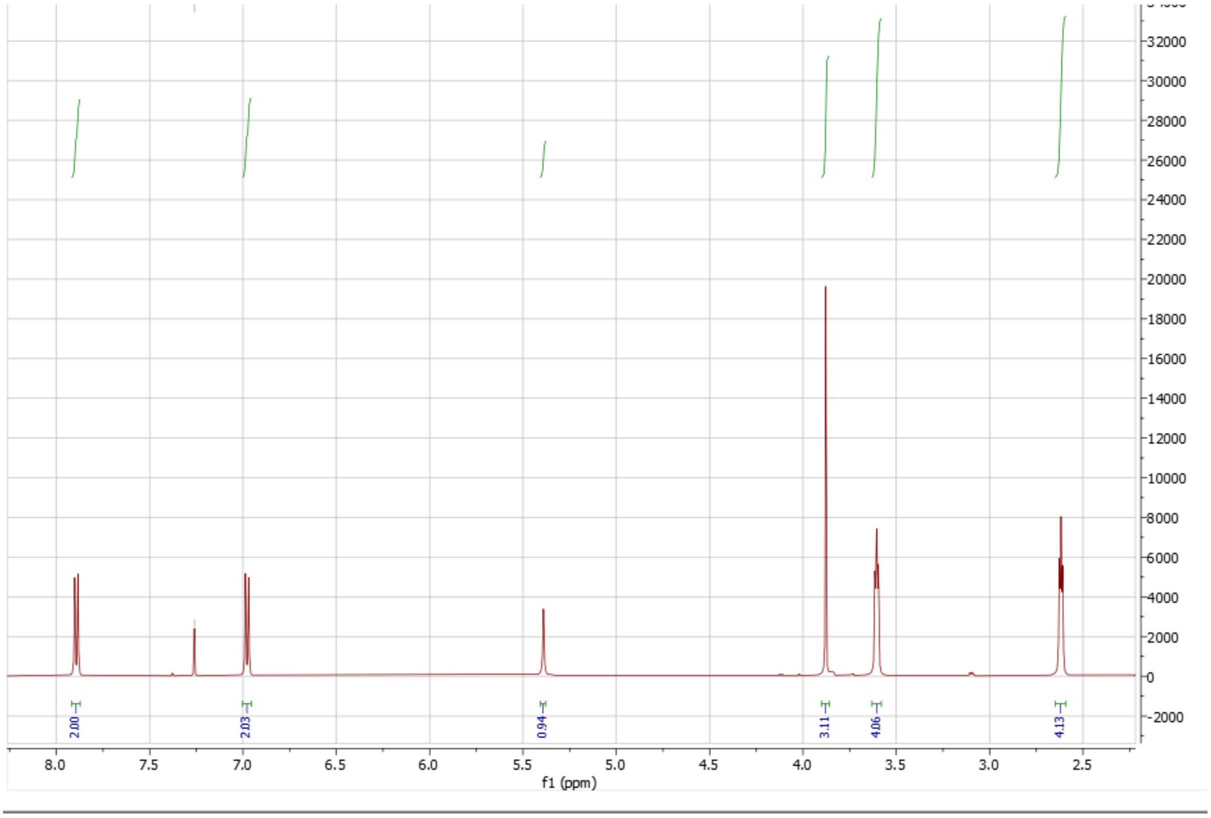

Figure S45. ^{1}H NMR spectrum of 4-methoxy-N-morpholinobenzenesulfonamide.

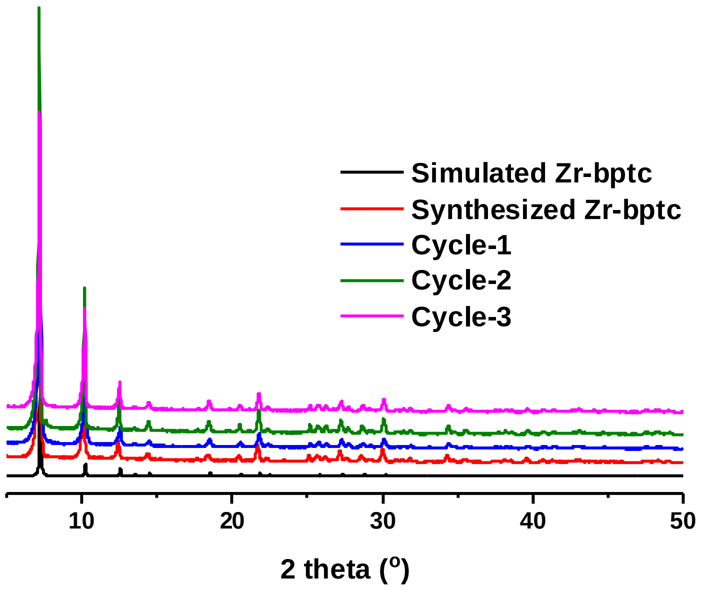

Figure S46. PXRD patterns for Zr-bptc after SO_2 conversion experiment.

Table S5. Elemental analysis and BET surface area on synthesized and recycled Zr-bptc

	Zr	C	H	BET (m^2/g)
Calculated	33.01	34.77	1.34	
Activated	32.73	33.43	1.50	960
Cycle-1	33.20	33.95	1.54	948
Cycle-2	32.95	33.48	1.37	950
Cycle-3	32.88	34.50	1.43	965

12. References

1. H. Wang, X. Dong, J. Lin, S. Teat, S. Jensen, J. Cure, E. Alexandrov, Q. Xia, K. Tan, Q. Wang, D. Olson, D. Proserpio, Y. Chabal, T. Thonhauser, J. Sun, Y. Han, J. Li, *Nat. Commun.* **2018**, *9*, 1-11.
2. J. Cavka, S. Jakobsen, U. Olsbye, N. Guillou, C. Lamberti, S. Bordiga, P. Lillerud, *J. Am. Chem. Soc.* **2008**, *130*, 13850–13851.
3. J. Garibay, M. Cohen, *Chem. Commun.* **2010**, *46*, 7700-7702.
4. K. Wang, H. Huang, W. Xue, D. Liu, X. Zhao, Y. Xiao, Z. Li, Q. Yang, L. Wang, C. Zhong, *CrystEngComm*, **2015**, *17*, 3586–3590.
5. Y. Ma, X. Han, S. Xu, Z. Wang, W. Li, I. Silva, S. Chansai, D. Lee, Y. Zou, M. Nikiel, P. Manuel, A. Sheveleva, F. Tuna, E. McInnes, Y. Cheng, S. Rudic, A. Ramirez-Cuesta, S. Haigh, C. Hardacre, M. Schröder, S. Yang, *J. Am. Chem. Soc.* **2021**, *143*, 10977-10985.
6. Y. Yan, A. O'Connor, G. Kanthasamy, G. Atkinson, D. Allan, A. Blake, M. Schröder, *J. Am. Chem. Soc.* **2018**, *140*, 3952-3958.
7. J. Hutter, M. Iannuzzi, F. Schiffmann, J. VandeVondele, *Wiley Interdiscip. Rev. Comput. Mol. Sci.* **2014**, *4*, 15-25.
8. G. Lippert, J. Hutter, M. Parrinello, *Mol. Phys.* **1997**, *92*, 477–487.
9. J. Vandevondele, K. Matthias, M. Fawzi, P. Michele, C. Thomas, H. Jurg, *Comput. Phys. Commun.* **2005**, *167*, 103–128.
10. J. VandeVondele, J. Hutter, *J. Chem. Phys.* **2007**, *127*, 114105.
11. S. Goedecker, M. Teter, J. Hutter, *Phys. Rev. B* **1996**, *54*, 1703-1710.
12. P. Perdew, K. Burke, M. Ernzerhof, *Phys. Rev. Lett.* **1996**, *77*, 3865–3868.
13. S. Grimme, J. Antony, S. Ehrlich, H. Krieg, *J. Chem. Phys.* **2010**, *132*, 154104.
14. Y. Cheng, L. Daemen, A. Kolesnikov, A. Ramirez-Cuesta, *J. Chem. Theory Comput.* **2019**, *15*, 1974–1982.
15. G. Smith, J. Eyley, X. Han, X. Zhang, J. Li, N. Jacques, H. Godfrey, S. Argent, L. McPherson, S. Teat. Y. Cheng, M. Frogley, G. Cinque, S. Day, C. Tang, T. Easun, S. Rudic, A. Ramirez-Cuesta, S. Yang, M. Schröder, *Nat. Mater.* **2019**, *18*, 1358-1365.
16. X. Cui, X. Wang, L. Yang, R. Krishma, Z. Zhang, Z. Bao, H. Wu, Q. Ren, W. Zhou, B. Chen, H. Xing, *Adv. Mater.* **2017**, *29*, 1606929.
17. S. Glomb, D, Woschko, G. Makhloufi, C. Janiak, *ACS Appl. Mater. Interfaces* **2017**, *9*, 37419-37434.
18. S. Yang, L. Liu, J. Sun, K. Thomas, A. Davies, M. George, A. Blake, A. Hill, A. Fitch, C. Tang, M. Schröder, *J. Am. Chem. Soc.* **2013**, *135*, 4954-4957.
19. K. Tan, P. Canepa, Q. Gong, J. Liu, D. Johnson, A. Dyevoich, P. Thallapally, T. Thonhauser, J. Li, Y. Chabal, *Chem. Mater.* **2013**, *25*, 4653-4662.
20. T. Grant Glover, G. Peterson, B. Schindler, D. Britt, O. Yaghi, *Chem. Eng. Sci.* **2011,** *66*, 163-170.
21. M. Savage, Y. Cheng, T. Easun, J. Eyley, S. Argent, M. Warren, W. Lewis, C. Murray, C. Tang, M. Frogley, G. Cinque, J. Sun, S. Rudic, R. Murden, M. Benham, A. Fitch, A. Blake, A. Ramirez-Cuesta, S. Yang, M. Schröder, *Adv. Mater.* **2016**, *28*, 8705-8711.
22. G. Lee, J. Lee, H. Vo, S. Kim, H. Lee, T. Park, *Sci. Rep.* **2017**, *7*, 557.
23. P. Thallapally, R. Motkuri, C. Fernandez, B. McGrail, G. Behrooz, *Inorg.Chem.* **2010**, *49*, 4909-4915.
24. C. Fernandez, P. Thallapally, R. Motkuri, S. Nue, J. Sumrak, J. Tian, J. Liu, *Cryst. Growth Des.* **2010**, *10*, 1037-1039.
25. J. Carter, X. Han, F. Moreau, I. Silva, A. Nevin, H. Godfrey, C. Tang, S. Yang, M. Schröder, *J. Am. Chem. Soc.* **2018**, *140*, 15564-15567.
26. L. Li, I. Silva, D. Kolokolov, X. Han, J. Li, G. Smith, Y. Cheng, L. Daemen, C. Morris, H. Godfrey, N. Jacques, X. Zhang, P. Manuel, M. Frogley, C. Murray, A. Ramirez-Cuesta, G. Cinque, C. Tang, A. Stepanov, S. Yang, M. Schröder, *Chem. Sci.* **2019**, *10*, 1472-1482.
27. M. Tchalala, P. Bhatt, K. Chappanda, S. Tavares, K. Adil, Y. Belmabkhout, A. Shkurenko, A. Cadiau, N. Heymans, G. Weireld, G. Maurin, K. Salama, M. Eddaoudi, *Nat. Commun.* **2019**, *10*, 1328.
28. E. Martínez-Ahumada, M. Diaz-Ramirez, H. Lara-Garcia, D. Williams, V. Martis, V. Jancik. E. Lima, I. Ibarra, *J. Mater. Chem. A.* **2020**, *8*, 11515-11520.
29. J. Zárate, E. Sanchez-Gonzalez, D. Williams, E. Gonzalez-Zamora, V. Martis, A. Martinez, J. Balmaseda, G. Maurin, I. Ibarra, *J. Mater. Chem. A.* **2019**, *7*, 15580-15584.

30. P. Brandt, A. Nuhnen, M. Lange, J. Mollmer, O. Weingart, C. Janiak, *ACS Appl. Mater. Interfaces* **2019**, *11*, 17350-17358.
31. L. Guo, X. Feng, Z. Gao, R. Krishna, F. Luo, *Inorg. Chem* **2021**, *60*, 1310-1314.
32. Z. Zhu, P. Zhang, B. Li, S. Chen, Q. Deng, Z. Zeng, J. Wang, S. Deng, *AIChE Journal* **2021**, *67*, e17300.
33. S. Valencia-Loza, A. Lopez-Olvera, E. Martinez-Ahumada, D. Martinez-Otero, I. Ibarra, V. Jancik, E. Percastegui, *ACS Appl. Mater. Interfaces* **2021**, *13*, 18658–18665.
34. Y. Fan, H. Zhang, M. Yin, R. Krishna, X. Feng, L. Wang, M. Luo, F. Luo, *Inorg. Chem.* **2021**, *60*, 4–8.
35. Y. Fan, M. Yin, R. Krishna, X. Feng, F. Luo, *J. Mater. Chem. A*, **2021**, *9*, 4075–4081.
36. M. Rivera-Almazo, M. Diaz-Ramirez, R. Hernandez-Esparza, R. Vargas, A. Martinez, V. Martis, P. Saenz-Cavazos, D. Williams, E. Lima, I. Ibarra, J. Garza, *Phys. Chem. Chem. Phys.* **2021**, *23*, 1454-1463.
37. S. Gorla, M. Diaz-Ramirez, N. Abeynayake, D. Kaphan, D. Williams, V. Martis, H. Lara-Garcia, B. Donnadieu, N. Lopez, I. Ibarra, V. Montiel-Palma, *ACS Appl. Mater. Interfaces* **2020**, *12*, 41758–41764.
38. Z. Chen, X. Wang, R. Cao, k. Idrees, X. Liu, M. Wasson, O. Farha, *ACS Materials Lett.* **2020**, *2*, 1129–1134.
39. J. Zarate, E. Dominguez-Ojeda, E. Sanchez-Gonzalez, E. Martinez-Ahumada, V. Lopez-Cervantes, D. Williams, V. Martis, I. Ibarra, J. Slejandre, *Dalton Trans.* **2020**, *49*, 9203–9207.
40. Y. Zhang, Z. Chen, X. Liu, Z. Dong, P. Zhang, J. Wang, Q. Deng, Z. Zeng, S. Zhang, S. Deng, *Ind. Eng. Chem. Res.* **2020**, *59*, 874−882.
41. Y. Zhang, P. Zhang, W. Yu, J. Zhang, J. Huang, J. Wang, M. Xu, Q. Deng, Z. Zeng, S. Deng. *ACS Appl. Mater. Interfaces* **2019**, *11*, 10680−10688.
42. Z. Zhang, B, Yang, Y. Wu, W. Zhang, H. Ma, *Separation and Purification Technology* **2021**, *276*, 119349.
43. J. Ponce, M. Diza-Ramirez, S. Gorla, C. Navarathna, G. Sanchez-Lecuona, B, Donnadieu, I. Ibarra, V. Montiel-Palma, *CrystEngComm* **2021**, *23*, 7479–7484.
44. Y. Sun, J. Liang, P. Brandt, A. Spieß, S. Ozturk, C. Janiak, *Nanoscale* **2021**, *13*, 15952–15962.
45. S. Wang, L. Chen, M. Wahiduzzaman, A. Tissot, L. Zhou, I. Ibarra, A. Gutierrez-Alejandre, J. Lee, J. Chang, Z. Liu, J. Marrot, W. Shepard, G. Maurin, Q. Xu, C. Serre. *Matter* **2021**, *4*, 182-194.
46. P. Brandt, S. Xing, J. Liang, G. Kurt, A. Nuhnen, O. Weingart, C. Janiak. *ACS Appl. Mater. Interfaces* **2021**, *13*, 29137–29149.
47. S. Xing, J. Liang, P. Brandt, F. Schafer, A. Nugnen, T. Heinen, I. Boldog, M. Lange, O. Weingart, C. Janiak. *Angew. Chem. Int. Ed.* **2021**, *60*, 17998–18005.
